# Mutant induced neurons and humanized mice enable identification of Niemann-Pick type C1 proteostatic therapies

**DOI:** 10.1172/jci.insight.179525

**Published:** 2024-10-22

**Authors:** Ruth D. Azaria, Adele B. Correia, Kylie J. Schache, Manuela Zapata, Koralege C. Pathmasiri, Varshasnata Mohanty, Dharma T. Nannapaneni, Brandon L. Ashfeld, Paul Helquist, Olaf Wiest, Kenji Ohgane, Qingqing Li, Ross A. Fredenburg, Brian S.J. Blagg, Stephanie M. Cologna, Mark L. Schultz, Andrew P. Lieberman

**Affiliations:** 1 Department of Pathology, University of Michigan Medical School, Ann Arbor, Michigan, USA.; 2 Department of Chemistry, University of Illinois Chicago, Illinois, USA.; 3 Department of Chemistry & Biochemistry and; 4 Warren Family Center for Drug Discovery, University of Notre Dame, Notre Dame, Indiana, USA.; 5 Department of Chemistry, Ochanomizu University, Tokyo, Japan.; 6 HitGen Inc, Chengdu, China.; 7 Ara Parseghian Medical Research Fund at Notre Dame University, Notre Dame, Indiana, USA.; 8 Stead Family Department of Pediatrics, Carver College of Medicine, University of Iowa, Iowa City, Iowa, USA.

**Keywords:** Neuroscience, Lysosomes, Neurological disorders, Protein misfolding

## Abstract

Therapeutics that rescue folding, trafficking, and function of disease-causing missense mutants are sought for a host of human diseases, but efforts to leverage model systems to test emerging strategies have met with limited success. Such is the case for Niemann-Pick type C1 disease, a lysosomal disorder characterized by impaired intracellular cholesterol trafficking, progressive neurodegeneration, and early death. NPC1, a multipass transmembrane glycoprotein, is synthesized in the endoplasmic reticulum and traffics to late endosomes/lysosomes, but this process is often disrupted in disease. We sought to identify small molecules that promote folding and enable lysosomal localization and functional recovery of mutant NPC1. We leveraged a panel of isogenic human induced neurons expressing distinct *NPC1* missense mutations. We used this panel to rescreen compounds that were reported previously to correct NPC1 folding and trafficking. We established mo56-hydroxycholesterol (mo56Hc) as a potent pharmacological chaperone for several NPC1 mutants. Furthermore, we generated mice expressing human I1061T NPC1, a common mutation in patients. We demonstrated that this model exhibited disease phenotypes and recapitulated the protein trafficking defects, lipid storage, and response to mo56Hc exhibited by human cells expressing I1061T NPC1. These tools established a paradigm for testing and validation of proteostatic therapeutics as an important step toward the development of disease-modifying therapies.

## Abstract 2

We report human iNeurons and mice expressing human I1061T NPC1, and we demonstrate their utility in identifying proteostatic therapies for Niemann-Pick type C disease.

## Introduction

Protein synthesis and folding in the endoplasmic reticulum (ER) is an iterative and highly regulated process, as nascent polypeptides undergo conformational changes and posttranslational modifications in an attempt to reach their functional, or native, conformation (1). Frequently, this process requires assistance from ER-resident chaperones that facilitate protein folding to enable subsequent export from the ER (2). These folding and trafficking processes govern a protein’s homeostasis, or “proteostasis.” However, commonly in human disease, missense mutations impair these processes. The resulting misfolded proteins are subject to degradation by ER quality control mechanisms, leading to the loss of functional proteins that underlies disease pathogenesis (2).

This cascade of events occurs in many individuals with Niemann-Pick type C1 disease, an autosomal recessive lysosomal disorder characterized by the accumulation of unesterified cholesterol in late endosomes and lysosomes (3). Disease course and severity are variable but include progressive neurodegeneration, hepatomegaly, and early death (4). Niemann-Pick type C1 disease is caused by mutations in the *NPC1* gene, which encodes a multipass transmembrane glycoprotein required for export of cholesterol from late endosomes and lysosomes (5). The disease hallmark of impaired intracellular lipid transport has many downstream consequences, including dysregulated ER calcium (6), disrupted autophagy (7–14), altered mTOR signaling (15, 16), lysosomal membrane permeabilization (16–18), and mitochondrial abnormalities (13, 19). Together, these result in myelination defects (20, 21) and neuron loss in the central nervous system (22–24).

Many of the more than 250 disease-causing mutations in *NPC1* are missense mutations that result in misfolded proteins that are either nonfunctional or degraded before reaching the lysosome (25). The latter is exemplified by the most common mutation in patients of western European ancestry, I1061T NPC1. Human I1061T NPC1 misfolds in the ER and is targeted to ER-associated degradation and ER-targeted macroautophagy (26–28). However, treatment with histone deacetylase inhibitors or ryanodine receptor antagonists corrects I1061T NPC1 folding and trafficking, enabling the protein to bypass ER quality control pathways and reach the lysosome, where it is functional (29, 30).

These observations have spurred interest in developing proteostatic therapeutics for Niemann-Pick type C1 disease, with the goal of rescuing folding and trafficking of the mutant protein to promote functional recovery. Notably, recent work has discovered significant differences in the trafficking of mouse versus human I1061T NPC1 that are attributable to differences in amino acid sequence (31). These distinctions affect the response of the mutant protein to proteostatic therapeutics and diminish the utility of *I1061T* *Npc1*–knockin mice for testing these compounds in vivo (32). Moreover, much prior work on proteostatic modulators used patient fibroblasts or other non-neuronal cell types that may have significantly different ER folding environments from primary neurons, a critical target cell in this disease.

To address these limitations, we utilized a panel of isogenic human induced pluripotent stem cell–derived (iPSC-derived) inducible neurons (iNeurons) with distinct *NPC1* disease-causing missense mutations. Here, we employed this panel to rescreen many of the small molecules that were reported previously to act as NPC1 proteostatic modulators. We established mo56-hydroxycholesterol (mo56Hc) (33, 34) as a potent pharmacological chaperone for several NPC1 missense mutants. Furthermore, we developed and characterized a mouse model expressing human I1061T NPC1. We demonstrated that this model exhibited critical disease phenotypes and recapitulated the trafficking defects, cholesterol storage, and response to mo56Hc exhibited by human cells expressing I1061T NPC1. These tools established a pipeline for testing and validation of NPC1 proteostatic therapeutics as an important step toward the development of disease-modifying therapies.

## Results

### Identification of an NPC1 proteostasis modulator using human iNeurons.

**Figure 1:**
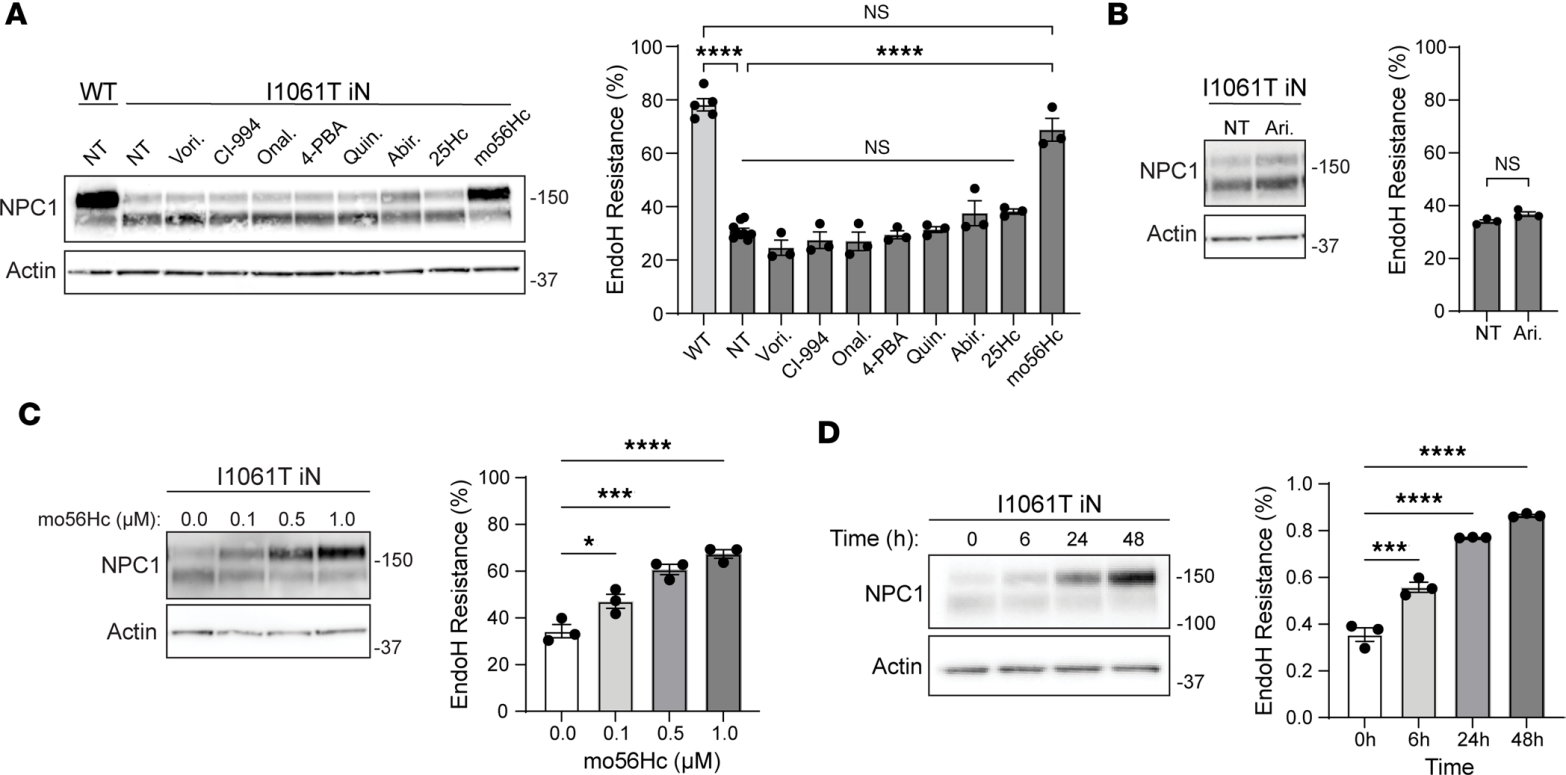
mo56Hc increases Endo H resistance of I1061T NPC1 in iNeurons. (**A** and **B**) Differentiated WT and I1061T NPC1 iNeurons were treated with vehicle (NT), 0.2 μM vorinostat (Vori.), 0.2 μM CI-994, 0.005 μM onalespib (Onal.), 100 μM 4-phenylbutyric acid (4-PBA), 10 μM quinestrol (Quin.), 10 μM abiraterone (Abir.), 1 μM 25-hydroxycholesterol (25Hc), 1 μM mo56-hydroxycholesterol (mo56Hc), or 400 μM arimoclomol (Ari.) for 48 hours. Lysates were digested with Endo H and analyzed by Western blot (values on right indicate ladder standard weights in kilodaltons). Quantified at right. (**C** and **D**) Differentiated I1061T NPC1 iNeurons were treated (**C**) for 48 hours with the indicated concentrations of mo56Hc or (**D**) with 1 µM mo56Hc for the indicated times.” Both experiments utilize mo56Hc treatment. Lysates were digested with Endo H and analyzed by Western blot. Quantified at right. All data are mean ± SEM from indicated number of independent experiments. *P ≤ 0.05, ***P ≤ 0.001, ****P ≤ 0.0001 by (**A**, **C**, and **D**) 1-way ANOVA with Tukey’s post hoc test or (**B**) *t* test. (**A**) *n* = 5 WT, 9 I1061T NPC1 NT, 3 I1061T NPC1 plus each drug; (**B**) *n* = 3 I1061T NPC1 NT, 3 I1061T NPC1 Ari.; (**C**) *n* = 3 I1061T NPC1 plus mo56Hc at each concentration; (**D**) *n* = 3 I1061T NPC1 plus mo56Hc for each treatment duration.

We sought to identify small molecules that promote trafficking of mutant NPC1 from the ER to late endosome/lysosomes to recover function in cell types that are critical targets of disease. To accomplish this, we leveraged a panel of isogenic iPSC-derived human iNeurons expressing wild-type (WT) or disease-causing *NPC1* missense mutations that were established using CRISPR/Cas9 gene editing. Cells expressing R1186H and P1007A NPC1 were generated to complement the recently reported human iNeurons expressing I1061T or R934L NPC1 (31). iPSCs with these mutations exhibited normal karyotypes (Supplemental Figure 1 and Supplemental Tables 1 and 2; supplemental material available online with this article; https://doi.org/10.1172/jci.insight.179525DS1) and pluripotency (Supplemental Figure 2). Following introduction of a human *Neurogenin-2* cassette, these cells were rapidly and efficiently differentiated into iNeurons after treatment with doxycycline (Supplemental Figure 3). In aggregate, this collection of isogenic iNeurons encompasses all 3 described classes of *NPC1* disease-causing missense mutations, including mutants that are largely retained in the ER (I1061T, R1186H), mutants that are found in both the ER and late endosomes/lysosomes (R934L), and mutants that traffic efficiently to late endosomes/lysosomes but have reduced function (P1007A) (25). Our studies used immature neurons, cultured 5 days after differentiation. Lysotracker staining showed significant lysosomal enlargement in iNeurons expressing I1061T and R1186H NPC1 (Supplemental Figure 3), correlating with limited trafficking of these mutants through the medial Golgi, as shown by endoglycosidase H (Endo H) assay (Figure 1).

iNeurons were used to examine the efficacy of compounds reported previously to promote the folding and trafficking of I1061T NPC1 in patient-derived fibroblasts as well as structurally similar small molecules. These compounds included the histone deacetylase inhibitors vorinostat (35) and CI-994 (35), the HSP90 inhibitor onalespib (36), and HSP modulator arimoclomol (37). These compounds are thought to act indirectly on I1061T NPC1, potentially through the regulation of molecular chaperones (25, 36–38). Other tested compounds included the chemical chaperone 4-phenylbutyric acid (27), as well as quinestrol (39), abiraterone (39), 25-hydroxycholesterol (25Hc) (33, 39), the previously described mo56Hc (33, 34), and U18666A (40). Although the mechanism of action of these small molecules is less well characterized, some may act as pharmacological chaperones (41) to stabilize folding intermediates (33, 39).

After identifying nontoxic doses using iNeurons (Supplemental Figure 4), we assessed these compounds for their effect on NPC1 trafficking through the medial Golgi by analysis of NPC1 glycan maturation. The simple high-mannose glycans added to NPC1 during synthesis and folding in the ER are sensitive to cleavage by the enzyme Endo H. However, Endo H is not able to cleave the complex glycans formed after NPC1 is processed in the medial and trans-Golgi. Thus, the sensitivity of glycans to Endo H cleavage indicates NPC1 trafficking relative to the Golgi.

**Figure 2:**
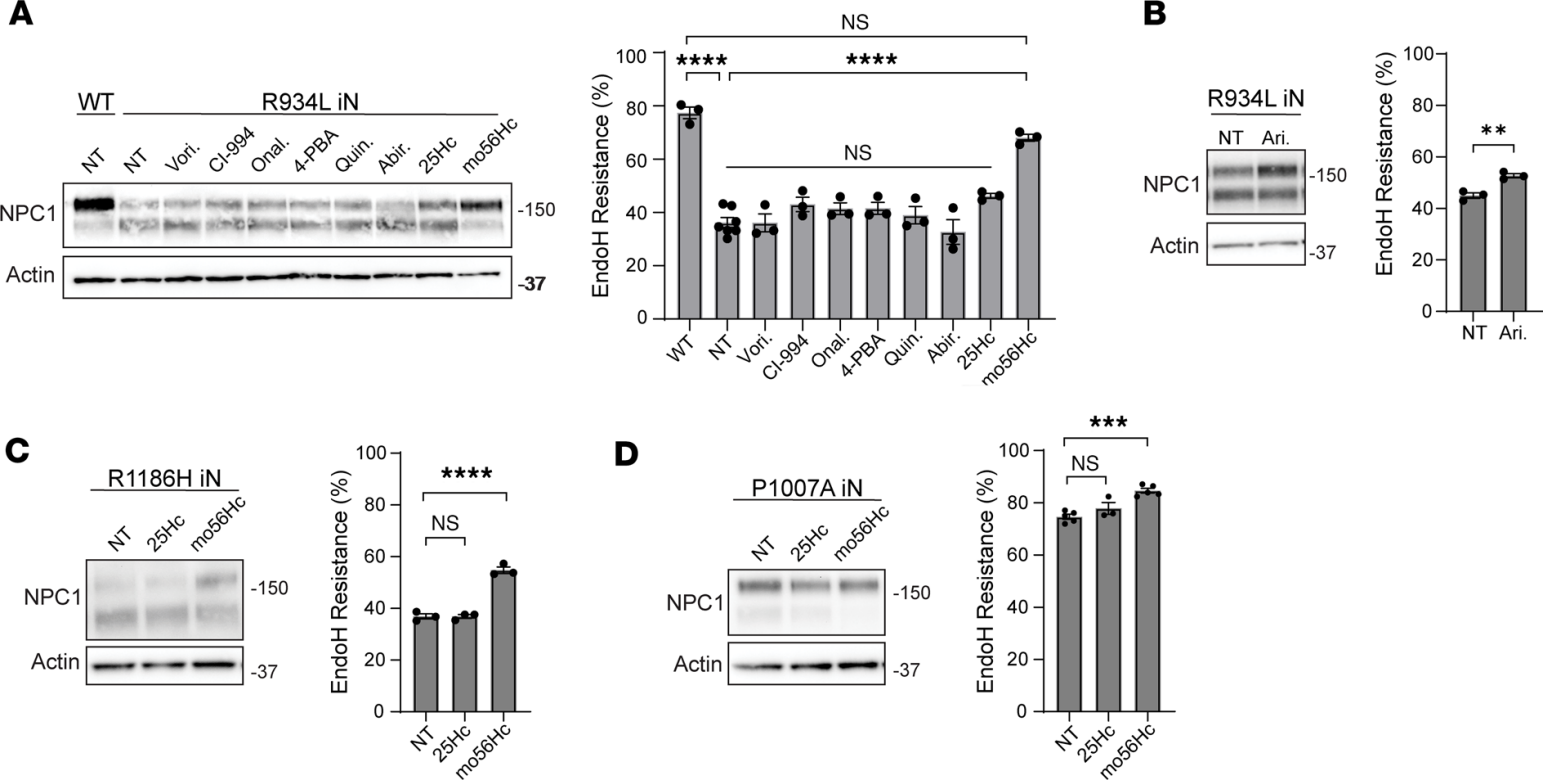
mo56Hc increases Endo H resistance of NPC1 trafficking mutants in iNeurons. (**A** and **B**) Differentiated WT and R934L NPC1 iNeurons were treated with vehicle (NT), 0.2 μM vorinostat (Vori.), 0.2 μM CI-994, 0.005 μM onalespib (Onal.), 100 μM 4-phenylbutyric acid (4-PBA), 10 μM quinestrol (Quin.), 10 μM abiraterone (Abir.), 1 μM 25-hydroxycholesterol (25Hc), 1 μM mo56-hydroxycholesterol (mo56Hc), or 400 μM arimoclomol (Ari.) for 48 hours. Lysates were digested with Endo H and analyzed by Western blot. Quantified at right. (**C** and **D**) Differentiated R1186H and P1007A iNeurons were treated with vehicle (NT), 1 μM 25Hc, or 1 μM mo56Hc for 48 hours. Lysates were digested with Endo H and subjected to Western blot. Quantified at right. All data are mean ± SEM from indicated number of independent experiments. **P ≤ 0.01, ***P ≤ 0.001, ****P ≤ 0.0001 by (**A**, **C**, and **D**) 1-way ANOVA with Tukey’s post hoc test or (**B**) *t* test. (**A**) *n* = 3 WT, 7 R934L NPC1 NT, 3 R934L NPC1 plus each drug; (**B**) *n* = 3 R934L NPC1 NT, 3 R934L NPC1 Ari.; (**C**) *n* = 3 R1186H NPC1 NT, 3 R1186H NPC1 plus each drug; (**D**) *n* = 3 P1007A NPC1 NT, 5 P1007A NPC1 plus each drug.

As anticipated, WT NPC1 was Endo H resistant, reflective of efficient trafficking through the Golgi to late endosomes/lysosomes (Figure 1A). In contrast, I1061T NPC1 exhibited Endo H sensitivity (Figure 1A), consistent with protein misfolding within the ER and impaired trafficking, as shown previously (31). Of the small molecule proteostatic modulators tested, only the modified hydroxycholesterol mo56Hc significantly increased the fraction of Endo H–resistant I1061T NPC1, suggesting improved trafficking (Figure 1, A and B, and Supplemental Figure 5). Similar effects were seen in human fibroblasts expressing I1061T NPC1 (Supplemental Figure 6B). Moreover, only mo56Hc significantly reduced lysosomal abundance and area as assessed by lysosomal-associated membrane protein 1 (LAMP1) staining in iNeurons expressing I1061T NPC1 (Supplemental Figure 6A), indicating an improvement in lysosomal pathology. The effects of mo56Hc on I1061T NPC1 Endo H resistance were both dose and time dependent (Figure 1, C and D). Similarly, vehicle-treated R934L NPC1 exhibited both Endo H–sensitive and –resistant species (Figure 2A). Of the modulators tested, mo56Hc significantly increased the fraction of Endo H–resistant R934L NPC1, demonstrating effects on multiple NPC1 trafficking mutants (Figure 2, A and B). Validating this conclusion, mo56Hc also significantly increased the Endo H resistance of R1186H NPC1 (Figure 2C). In contrast, relatively minor effects were seen on the fraction of Endo H–resistant P1007A NPC1, a mutant that traffics efficiently to late endosome/lysosomes but is functionally impaired (Figure 2D). Together, these data suggest that mo56Hc robustly corrects the trafficking of ER-retained NPC1 mutants in iNeurons and fibroblasts.

**Figure 3:**
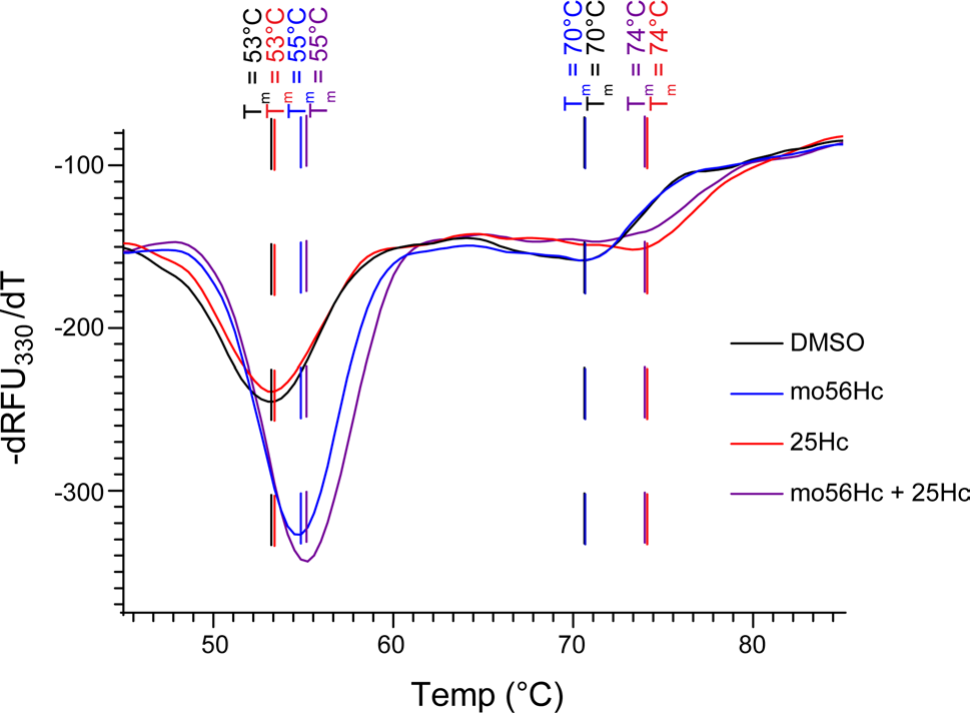
m56Hc increases thermal stability of NPC1 protein. Purified WT NPC1 protein was treated with 50 μM mo56Hc (blue lines and text), 50 μM 25Hc (red lines and text), or 50 μM mo56Hc + 50 μM 25Hc (purple lines and text) before thermal stability analysis was performed via monitoring of inherent tryptophan fluorescence using nano-differential scanning fluorimetry. First derivative analysis of the 330 nm emission wavelength is shown. The stabilization of NPC1 by mo56Hc is apparent in a shift in Tm as well as an increase in slope of the thermal transition.

To glean insights into this compound’s mechanism of action, direct effects of mo56Hc and 25Hc, a structurally similar but inactive oxysterol, were examined on purified NPC1 protein using a thermal stability assay. NPC1 unfolds in 2 distinct thermal transitions as seen in this assay (Figure 3). The 2 tested compounds had distinct effects on NPC1 thermal stability. 25Hc shifted the melting temperature of the later thermal transition by 3°C to the right, while mo56Hc shifted both the melting temperature and slope of the early thermal transition; both effects are known to indicate stabilization of a protein (42). Combined treatment with 25Hc and mo56Hc stabilized both thermal transitions, suggesting that these structurally related molecules do not cross-react between their respective binding sites on the NPC1 protein. Notably, mo56Hc stabilized NPC1 at concentrations well below 50 μM, displaying an EC_50_ of approximately 3 μM in the thermal stability assay (Supplemental Figure 8). Microscale thermophoresis showed mo56Hc binds NPC1 with an EC_50_ of 177 nM (Supplemental Figure 8). These data are compatible with activity in cellular assays in the high nanomole to low micromole concentration range, as shown in Figure 1. These analyses support a model in which mo56Hc stabilizes NPC1 in a conformation that favors trafficking.

### Development and characterization of humanized I1061T NPC1 mice.

**Figure 4:**
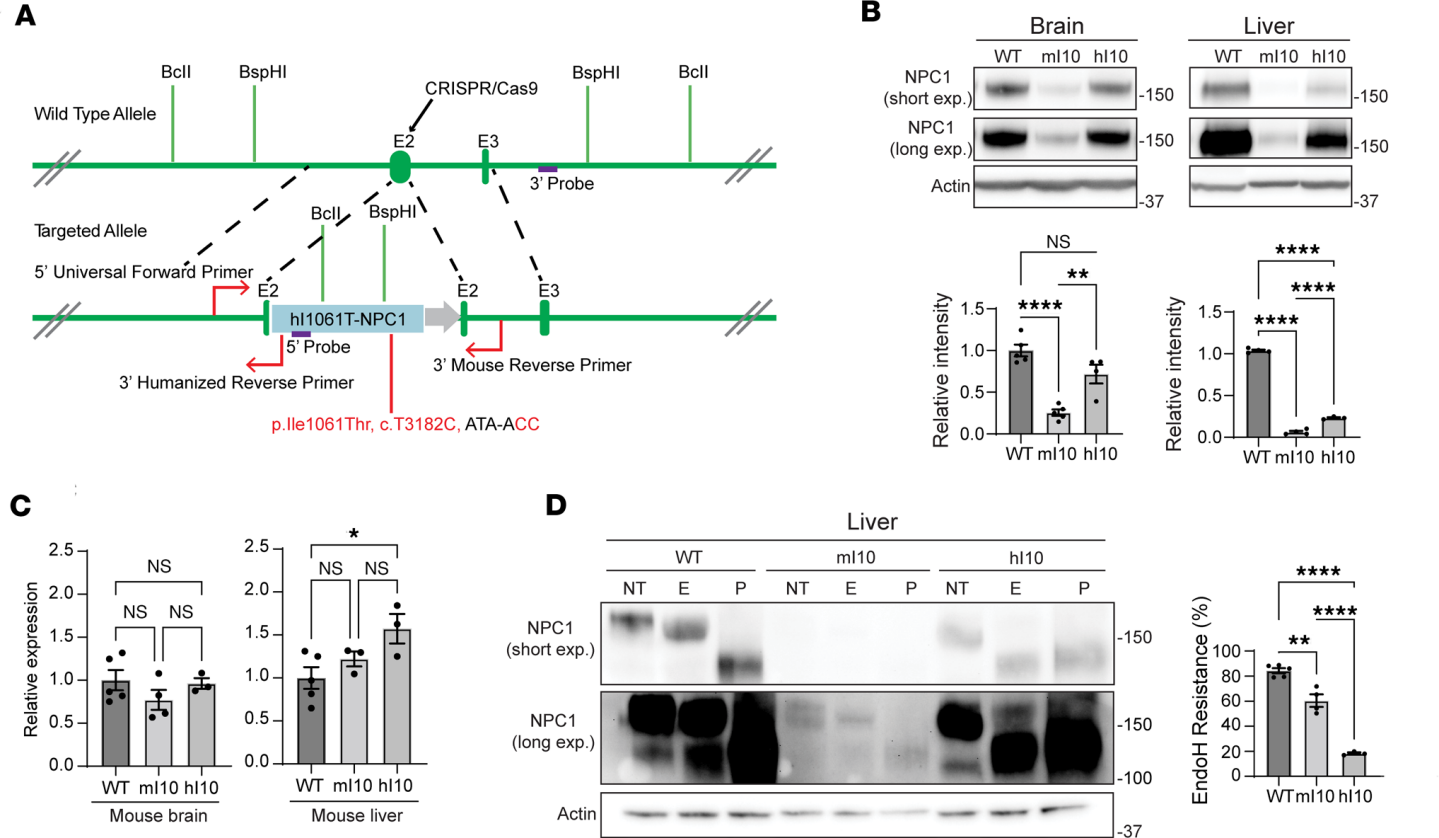
Generation of humanized I1061T NPC1 mice. (**A**) Depiction of the editing strategy used to create the humanized I1061T NPC1 mouse. The human *NPC1* cDNA sequence encoding the I1061T NPC1 mutation was inserted into exon 2 of mouse *Npc1*, downstream of the signal peptide sequence, by CRISPR/Cas9 homology-directed repair. Restriction enzymes (BcII, BspHI) and Southern blot (5′ and 3′ probes) were used to determine proper insertion and PCR (5′ universal forward primer, 3′ humanized reverse primer, 3′ mouse reverse primer) were designed for mouse genotyping. (**B**) Brain and liver tissue from 6- to 8-week-old WT, knockin mouse I1061T NPC1 (mI10), and humanized I1061T NPC1 (hI10) were analyzed for total NPC1 protein by Western blot. Quantified below. (**C**) Brain and liver tissue from WT, mI10, and hI10 mice were analyzed for NPC1 mRNA by qPCR. (**D**) Liver lysates from WT, mI10, and hI10 mice were digested with Endo H and subjected to Western blot. Quantified at right. NT, not treated; E, Endo H digested; P, PNGase F digested. All data are mean ± SEM from the indicated number of independent experiments. *P ≤ 0.05, **P ≤ 0.01, ****P ≤ 0.0001 by (**B**–**D**) 1-way ANOVA with Tukey’s post hoc test. (**B**) For brain, *n* = 5 WT, 5 mI10, 4 hI10; for liver, *n* = 4 WT, 4 mI10, 3 hI10. (**C**) For brain, *n* = 5 WT, 4 mI10, 3 hI10; for liver, *n* = 5 WT, 3 mI10, 3 hI10; (**D**) *n* = 5 WT, 4 mI10, 3 hI10.

We next sought to establish a mouse model for testing small molecules that rescue NPC1 trafficking to late endosomes/lysosomes. We recently demonstrated the unanticipated observation that mouse I1061T NPC1 does not accurately model the folding and trafficking defects of human I1061T NPC1 because of species-specific differences in protein sequence (31). To create a mouse model that recapitulates the protein defects of human I1061T, we used CRISPR/Cas9 gene editing to insert the full-length human *I1061T NPC1* cDNA into exon 2 of the mouse *Npc1* gene (Figure 4A). This targeting strategy enabled insertion of human coding sequence immediately after the mouse signal peptide, thereby retaining endogenous gene regulatory elements upstream of exon 2. Successful gene editing was confirmed by Southern blot (Supplemental Figure 9A).

Mice expressing human I1061T NPC1 (referred to as humanized I1061T mice, abbreviated hI10) were bred to homozygosity, and NPC1 expression was compared with WT and previously established mouse I1061T-knockin mice (abbreviated mI10) (32). Western blot of brain and liver lysates demonstrated significantly higher levels of NPC1 protein in hI10 than mI10 mice (Figure 4B). Quantitative real-time PCR (qPCR) revealed similar levels of *NPC1* mRNA in these mice and in WT controls, suggesting that the mouse I1061T NPC1 protein was more efficiently degraded than the mutant human protein (Figure 4C). Importantly, Endo H assay established that I1061T NPC1 trafficking defects in hI10 mice modeled what is seen in human cells (Figure 4D and Supplemental Figure 9B) (31). While most mouse WT NPC1 expressed in liver and brain was Endo H resistant, human I1061T NPC1 was predominantly Endo H sensitive. In contrast, mouse I1061T NPC1 was largely Endo H resistant.

**Figure 5:**
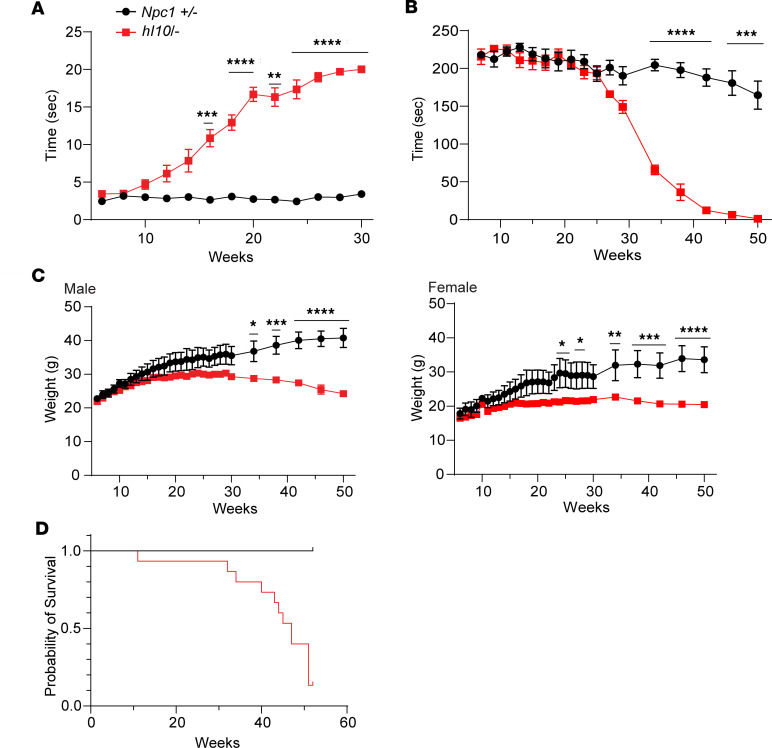
Humanized I1061T NPC1 mice exhibit age-dependent motor impairment, diminished body weight, and decreased survival. (**A**) Mice were tested for their average time to cross a balance beam at the indicated weeks of age. (**B**) Mice were tested for their average time spent running on an accelerating rotarod at the indicated weeks of age. (**C**) Body weights were measured at the indicated weeks of age. Males, left; females, right. (**D**) Survival probability of tested mice was calculated over time. *P* = 0.0003 by log-rank test. (**A**–**D**) *Npc1^+/–^* in black; *hI10^/–^* in red. All data are shown as mean ± SEM. *P ≤ 0.05, **P ≤ 0.01, ****P ≤ 0.0001 by (**A**–**D**) 2-way ANOVA with Bonferroni’s post hoc test. (**A**, **B**, and **D**) *n* = 8 *Npc1^+/–^*, 12 *h10^/–^*; (**C**) *n* = 4–5 males/group, *n* = 3–6 females/group.

To characterize the phenotype of hI10 mice, a cohort of C57BL/6J and BALB/cJ F1 hybrids were generated carrying 1 hI10 allele (on C57BL/6J background) and 1 null allele (on BALB/cJ background). This strategy was used here since it is known to significantly increase the yield of homozygous mutant mice in other mouse models of Niemann-Pick C1 disease. The resulting experimental mice (*hI10^/–^*) and littermate controls (*Npc1^+/–^*) were aged to 52 weeks. Mice expressing the hI10 allele developed age-dependent motor impairment as measured by balance beam and rotarod performance (Figure 5, A and B), diminished body weight in both males and females (Figure 5C), and impaired survival (Figure 5D).

**Figure 6:**
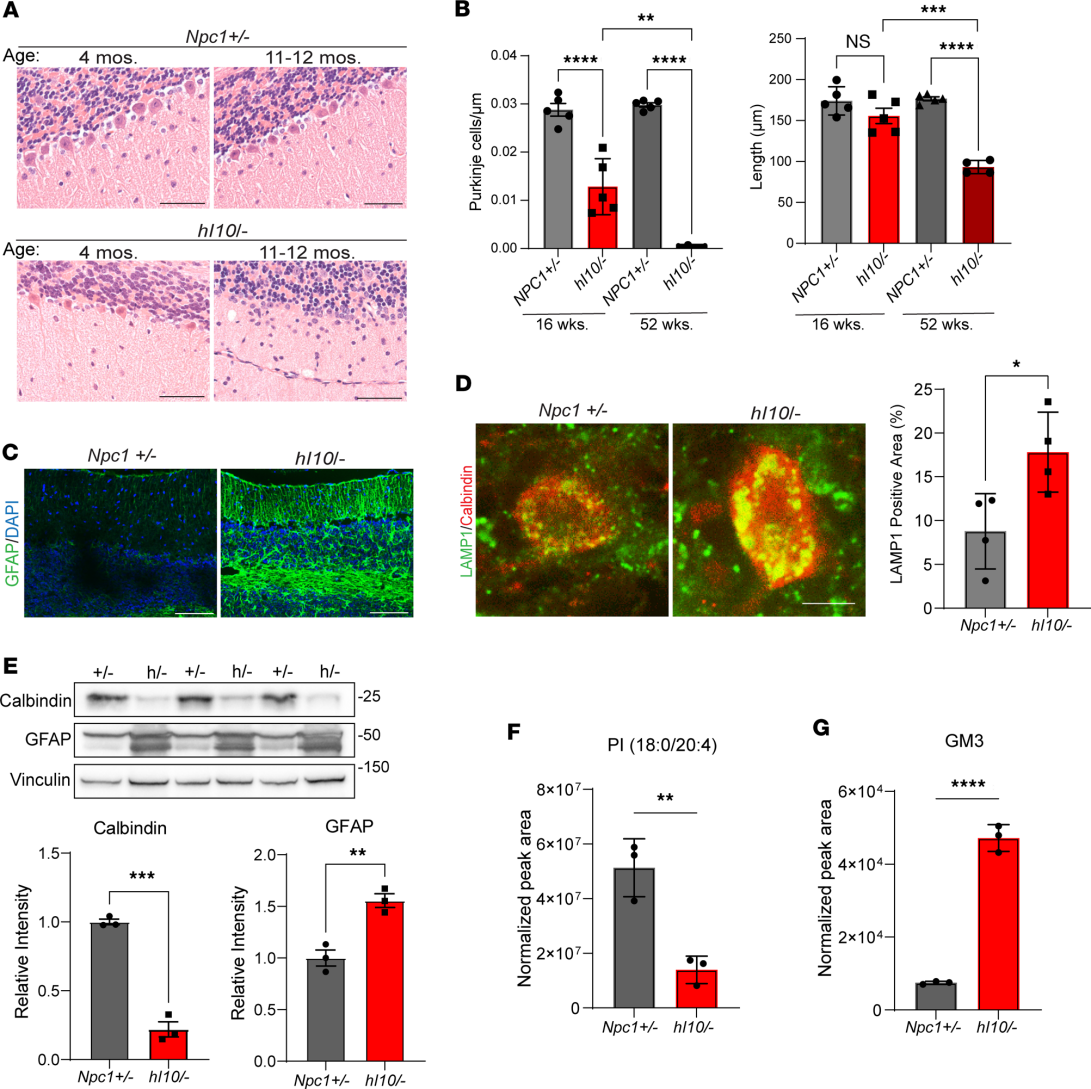
Age-dependent neurodegeneration in humanized I1061T NPC1 mice. (**A**) Midline sagittal sections of cerebellar lobules V–VI were stained with H&E and imaged. Top, *Npc1^+/–^*; bottom, *hI10^/–^*. Scale bar = 50 μm. (**B**) Purkinje cell count and molecular layer thickness in cerebellar lobules V–VI were quantified in *Npc1^+/–^* and *hI10^/–^* mice at 16 and 52 weeks of age. (**C**) The cerebellums of 16-week-old mice were stained for GFAP (green). Nuclei stain by DAPI (blue). Scale bar = 100 μm. (**D**) Purkinje neurons in the cerebellum of 52-week-old mice were stained for calbindin (red) and LAMP1 (green). LAMP1-positive area quantified at right. Scale bar = 10 μm. (**E**) Cerebellar lysates from 52-week-old *Npc1^+/–^*(+/-) and *hI10+* (h/-) mice were probed for calbindin and GFAP. Quantified below. (**F** and **G**) Mass spectrometry lipidomic analysis of hindbrain from *Npc1^+/–^* and *hI10^/–^* at 52 weeks showed a significant (**F**) decrease of phosphatidylinositol (PI 18:0/20:4) and (**G**) increase of ganglioside GM3 (d18:1/20:0) in mutant mice. **B**, **D**, and **E** are shown as mean ± SEM. **F** and **G** are shown as mean ± SD. *P ≤ 0.05, **P ≤ 0.01, ***P ≤ 0.001, ****P ≤ 0.0001 by (**B**) 2-way ANOVA with Bonferroni’s post hoc test or (**D**–**G**) *t* test. (**B**) At 16 weeks *n* = 5/group; at 52 weeks, *n* = 4–5/group; (**D**) *n* = 4 mice/genotype; (**E**–**G**) *n* = 3/group.

**Figure 7:**
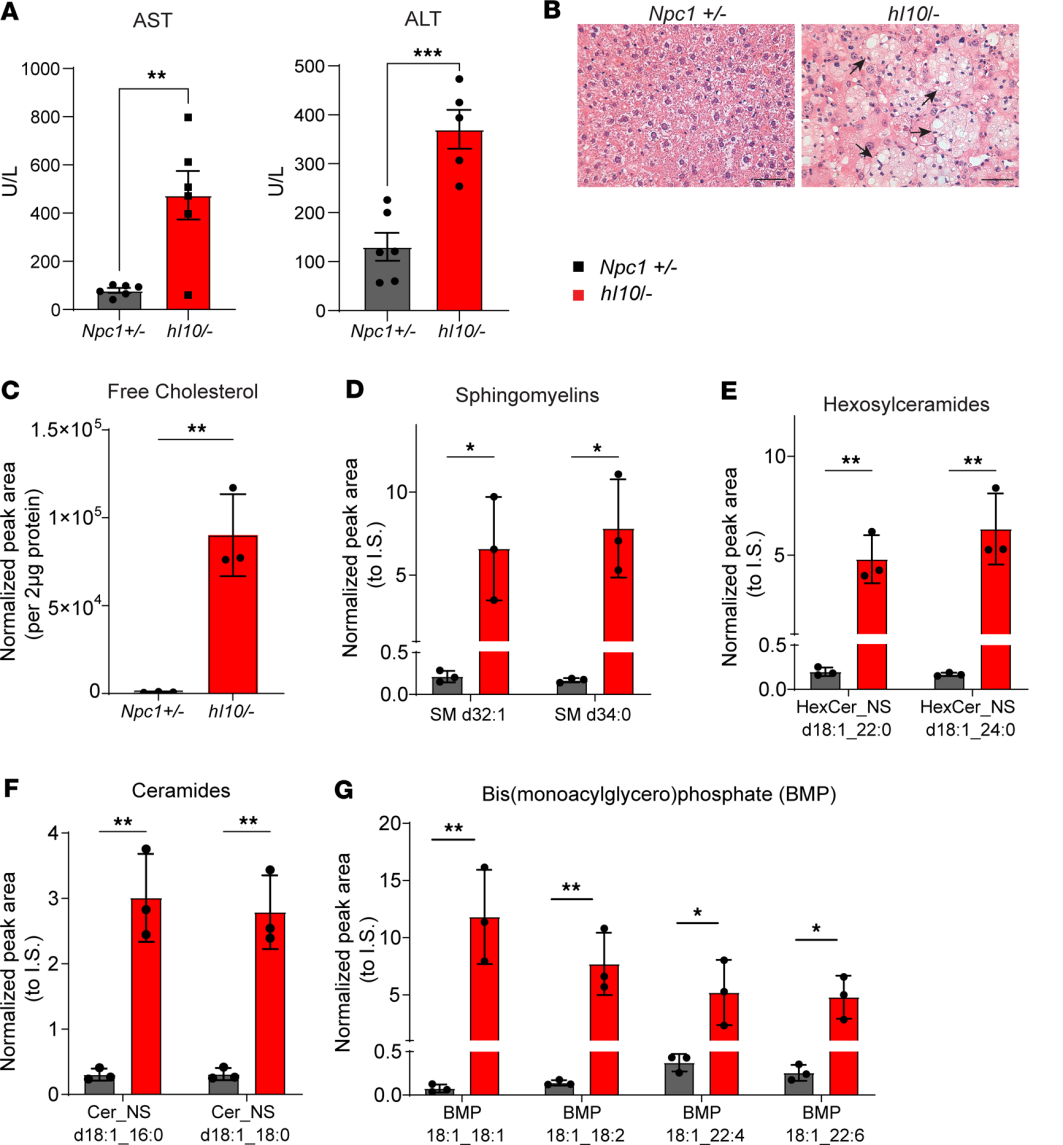
Liver pathology in humanized I1061T NPC1 mice. (**A**) Serum AST and ALT were measured in mice at 52 weeks. U/L, units/liter. (**B**) Liver from 52-week-old *Npc1^+/–^* and *hI10^/–^* mice were stained with H&E and imaged. Arrows highlight clusters of foamy macrophages. Scale bar = 50 μm. (**C**–**G**) Mass spectrometry lipidomic analysis of liver tissue from *Npc1^+/–^* and *hI10^/–^* at 52 weeks showed significant increases of (**C**) free cholesterol, (**D**) sphingomyelins (SM), (**E**) hexosylceramides (HexCer), (**F**) ceramides (Cer), and (**G**) bis(monoacylglycero)phosphate (BMP) in mutant mice. (**D**–**G**) *Npc1^+/–^* in black; *hI10^/–^* in red. (**A**) Mean ± SEM. (**C**–**G**) Mean ± SD. *P ≤ 0.05, **P ≤ 0.01, ***P ≤ 0.001 by (**A** and **C**–**G**) 2-tailed *t* test. (**A**) *n* = 6/group, (**C**–**G**) *n* = 3/group. I.S., internal standard.

Histopathological analysis demonstrated that mutant mice developed age-dependent cerebellar degeneration characterized by the progressive loss of Purkinje neurons and thinning of the molecular layer of cerebellar folia (Figure 6, A and B). This was accompanied by the accumulation of unesterified cholesterol in the soma of Purkinje neurons as highlighted by filipin staining (Supplemental Figure 10) and by the presence of reactive astrocytes (Figure 6C) that were detectable as early as 16 weeks. By 52 weeks, the soma of Purkinje neurons showed a significant increase in size of LAMP1-positive vesicles (Figure 6D). Western blot of cerebellar lysates showed significant loss of the Purkinje neuron marker calbindin and accumulation of glial fibrillary acidic protein (GFAP), a marker of reactive astrocytes (Figure 6E). Additionally, Western blot showed aberrant, decreased mobility of LAMP1 (Supplemental Figure 10), identical to changes previously reported in *Npc1*-null mice because of aberrant glycosylation (43). Mass spectrometry–based lipidomic analysis identified numerous alterations (Supplemental Table 3), including significantly decreased phosphatidylinositol and accumulation of the ganglioside GM3 (monosialodihexosylganglioside) (d18:1/20:0) in hindbrain lysates (Figure 6, F and G). Moreover, mutant mice at 52 weeks had elevated levels of serum aspartate transaminase (AST) and alanine transaminase (ALT) (Figure 7A), along with the accumulation of foamy macrophages in the liver (Figure 7B). These changes were associated with the significant accumulation of numerous lipids (Supplemental Table 3), including free cholesterol, sphingomyelin, hexosylceramides, ceramides, and bis(monoacylglcero)phosphate (BMP) in liver (Figure 7, C–G). These data demonstrated that humanized I1061T NPC1 mice developed age-dependent behavioral phenotypes and histopathology similar to *Npc1*-null mice (23) but that these occurred with a delayed time course.

### Cells from humanized I1061T NPC1 respond to mo56Hc.

**Figure 8:**
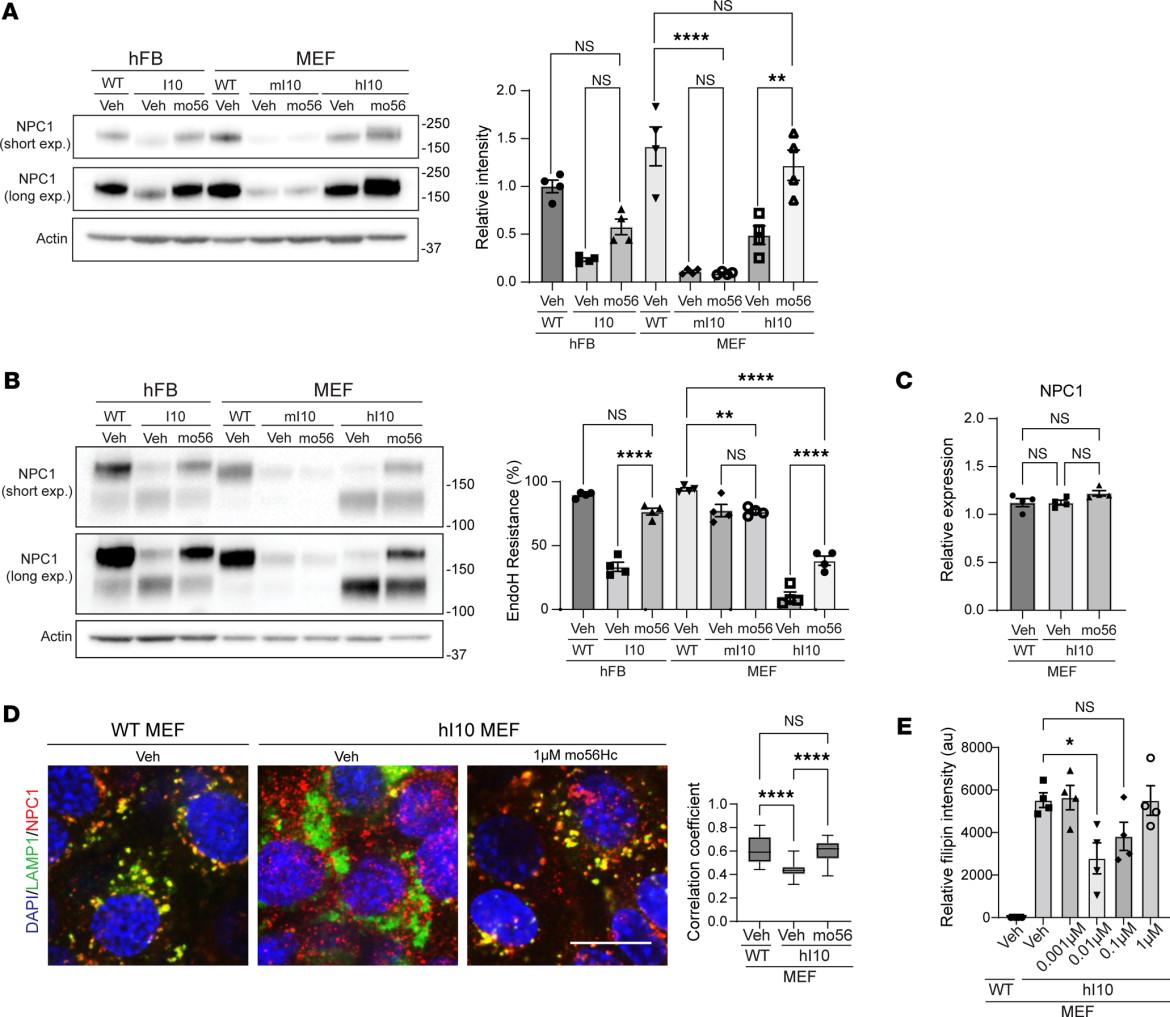
mo56Hc rescues humanized I1061T NPC1 trafficking, localization, and function. (**A** and **B**) Human fibroblasts (hFB) expressing WT or I1061T NPC1 (I10) and mouse fibroblasts (MEFs) expressing mouse I1061T NPC1 (mI10) or human I1061T NPC1 (hI10) were treated with 1 μM mo56Hc for 48 hours. (**A**) Total NPC1 was analyzed by Western blot. Quantified at right. (**B**) Lysates were digested with Endo H and subjected to Western blot. Quantified at right. (**C**) MEFs expressing mouse WT or human I1061T NPC1 were treated with vehicle or 1 μM mo56Hc for 48 hours and subjected to qPCR for NPC1 gene expression. (**D**) MEFs expressing mouse WT or human I1061T NPC1 were treated with vehicle or 1 μM mo56Hc for 48 hours. Cells were stained for NPC1 (red) and LAMP1 (green). Nuclei were stained with DAPI (blue). Scale bar = 10 μm. Colocalization of NPC1 and LAMP1 is quantified to right. (**E**) MEFs expressing mouse WT or human I1061T NPC1 were treated with vehicle or varying doses of mo56Hc for 48 hours. Unesterified cholesterol was labeled with filipin and quantified. All data are mean ± SEM from the indicated number of independent experiments. *P ≤ 0.05, **P ≤ 0.001, ****P ≤ 0.0001 by (**A**–**E**) 1-way ANOVA with Tukey’s post hoc test. (**A**–**C**) *n* = 4 for all groups. (**D**) The box encompasses the 25th to 75th percentile of each data set, the horizontal bar indicates the data set median, and the whiskers represent min and max values for the data set. *n* = 33 WT MEF images, 32 hI10 MEF Veh images, 32 hI10 MEF mo56Hc images. (**E**) *n* = 4 for all groups, with each *n* representing an average of 8 images per data point.

To assess the response of humanized I1061T NPC1 to proteostasis modulators, we developed a line of immortalized fibroblasts from the tail of homozygous hI10 mice. These cells were compared with human fibroblasts expressing WT or I1061T NPC1, as well as mouse fibroblasts expressing WT or mouse I1061T NPC1. Following treatment with vehicle or mo56Hc, lysates were assessed for total NPC1 levels and Endo H resistance (Figure 8, A and B). mo56Hc significantly increased total NPC1 levels in fibroblasts from hI10 mice (Figure 8A) without altering *NPC1* mRNA expression (Figure 8C). This was associated with a significant increase in the fraction of Endo H–resistant human I1061T NPC1 in hI10 mouse and human fibroblasts (Figure 8B). No additive effect on Endo H resistance was observed following treatment with both mo56Hc and 25Hc (Supplemental Figure 11). Notably, mo56Hc had no effect on the total level or Endo H resistance of mouse I1061T NPC1 (Figure 8, A and B), supporting the conclusion that species-specific differences in I1061T NPC1 folding and trafficking affected the response to small molecule proteostasis modulators.

To verify that mo56Hc rescued lysosomal localization of I1061T NPC1, we quantified colocalization of NPC1 with the lysosomal marker LAMP1 (Figure 8D). This analysis demonstrated significantly diminished colocalization of human I1061T NPC1 compared with WT NPC1 and rescue following treatment with mo56Hc. Improved localization of I1061T NPC1 following mo56Hc treatment yielded functional rescue, as demonstrated by staining with filipin, a fluorescent dye that binds unesterified cholesterol (Figure 8E and Supplemental Figure 7). Quantification demonstrated a U-shaped concentration response curve, similar to that reported previously in human fibroblasts (33). Together, these data demonstrated that hI10 fibroblasts responded to mo56Hc by trafficking to the lysosome and clearing cholesterol, indicating that the new hI10 mouse model will be an effective tool for in vivo testing of proteostasis modulators.

## Discussion

The degradation of misfolded mutant proteins by ER quality control pathways underlies the pathogenesis of a host of inherited human disorders. Efforts to identify proteostatic therapeutics focus on the idea that some of these mutant proteins retain function if properly folded and trafficked. Studies aimed at leveraging mouse model systems to develop these strategies have met with limited success, in part because of underlying species differences that affect proteostasis. Here, we report the development of model systems for the testing and identification of compounds that promote folding, trafficking, and functional recovery of missense mutants that cause Niemann-Pick type C1 disease. This inherited lysosomal disorder is caused by loss-of-function mutations in the *NPC1* gene. Efforts to develop disease-modifying therapies are aimed at mitigating the progressive neurological impairment that invariably leads to early death, a significant unmet need for patients with Niemann-Pick C1.

We used CRISPR/Cas9 gene editing to generate a suite of isogenic human iNeurons with 4 distinct *NPC1* missense mutations that occur in Niemann-Pick C1. These cell lines include representative missense mutations capturing the range of effects on NPC1 trafficking that occur in individuals with disease (25). Human iNeurons were selected as a model because of the critical role neurons play in the neurodegenerative pathology (22–24). As proof of concept, we tested 9 small molecules that have been reported in the literature as beneficial to mutant NPC1 proteostasis in human fibroblasts. Only a single compound, the modified hydroxycholesterol mo56Hc, exhibited significant activity in our systems by promoting trafficking of mutant NPC1 through the medial Golgi. Interestingly, mo56Hc similarly rescued 3 distinct ER-retained NPC1 mutants, I1061T, R934L, and R1186H, but had little effect on P1007A, a mutant that efficiently traffics to late endosomes/lysosomes. These data support a model in which mo56Hc binds nascent NPC1 in the ER and stabilizes a protein conformation that traffics appropriately. This model is supported by thermal stability studies, as well as by data demonstrating I1061T NPC1 colocalization with lysosomal markers and rescue of stored cholesterol following treatment with mo56Hc.

We also report the generation and characterization of mice expressing human I1061T NPC1, the most common missense mutation found in patients of western European ancestry. This model exhibits disease phenotypes similar to previously characterized NPC1-mutant mice. Importantly, the human I1061T NPC1 protein expressed in these mice recapitulates the trafficking defects occurring in human cells and responds to treatment with mo56Hc. These features distinguish this model from the previously characterized mouse *I1061T Npc1* knockin (32) and demonstrate its utility for testing proteostatic therapies.

The notable differences between mouse and human I1061T NPC1 underscore the importance of humanized mice for analyzing compounds that affect protein folding and trafficking. Although *NPC1* mRNA levels are similar in knockin and humanized mice (Figure 4C), mouse I1061T NPC1 protein levels are significantly lower (Figure 4B), suggesting that most of the mouse protein is rapidly degraded. This is recapitulated in mouse fibroblasts, where mouse I1061T NPC1 protein is expressed at lower levels than human I1061T NPC1 protein (Figure 8A). In tissue from humanized I1061T mice, diminished NPC1 protein levels are more evident in liver than brain (Figure 4B), raising the additional possibility that there exist tissue-specific differences in its degradation. Importantly, only human I1061T NPC1 protein responds to treatment with mo56Hc by demonstrating increased trafficking through the Golgi (Figure 8, B and D). We suggest that the majority of mouse I1061T NPC1 is in an unfolded state that prevents binding of mo56Hc, leading to it being refractory to the compound’s beneficial effects and triggering rapid degradation of the protein. In contrast, human I1061T NPC1, while unfolded, appears more stable and is in a conformation that is permissive to mo56Hc binding and therapeutic benefits.

The action of mo56Hc as a pharmacologic chaperone for NPC1 is intriguing, and the thermal stability analysis offers insights into its mechanism of action (Supplemental Figure 12). Given the published interaction of 25Hc with the NPC1 N-terminal domain (NTD) (44), it is likely that the second thermal transition in NPC1 represents the unfolding of the NTD. This suggests that the early thermal transition represents denaturation of the remainder of the protein, including the transmembrane (TM) domain composed of 12 helical segments (45). Within the TM domain lies the sterol-sensing domain (SSD), a structurally conserved cholesterol binding domain that serves as a potential binding site for mo56Hc (46, 47). Mo56Hc is likely excluded from the cholesterol site within the NTD due to the modification of the 3β-hydroxyl group, which is vital to cholesterol binding (44). Supporting this, mutation of the NPC1 SSD reduces the binding of mo56Hc derivative, while binding of mo56Hc to NPC1 is preserved in the absence of the NTD (48). The direct effects observed in our studies are consistent with the published properties of these molecules and further verify that ligands of NPC1 stabilize the protein.

The distinction between mo56Hc and 25Hc may explain the phenotypic differences of their activity in cells. Stabilization of only the NTD may not address the global instability of the I1061T NPC1 variant; this mutation is localized to the C-terminal domain, within the portion of the protein that likely unfolds in the early thermal transition displayed in Figure 3. The likelihood that mo56Hc stabilizes the core of the protein, which is disrupted by the I1061T mutation and many other pathogenic NPC1 mutations, may indicate a more productive effect on the protein’s processing and function within the cell. The sparing of the NTD by mo56Hc avoids competition with cholesterol, a known effect of 25Hc (49), as supported by the functional rescue of mo56Hc-treated I1061T NPC1 (Figure 8E). These findings highlight important features that guide the further development of pharmacologic chaperones for NPC1. Moreover, our analyses demonstrate the utility of the model systems reported here and advance efforts to generate disease-modifying therapies for patients with Niemann-Pick type C1.

## Methods

Further information appears in Supplemental Methods.

### Sex as a biological variable

Approximately equal numbers of male and female mice were used in these studies.

### Antibodies

#### Primary antibodies.

The following primary antibodies (antigen, dilution, vendor) were used: NPC1, 1:500, Abcam ab134113/clone EPR5209; Actin, 1:2,000, MilliporeSigma A5441/clone AC-15; Map2, 1:150, MilliporeSigma MAB3418/clone AP20; LAMP1, 1:50, Developmental Studies Hybridoma Bank University of Iowa 1D4B; Calbindin, 1:2,000, MilliporeSigma C2724/clone EG-20; GFAP, 1:500, Dako Z0334; and Vinculin, 1:2,000, MilliporeSigma V9131/clone hVIN-1.

#### Secondary antibodies.

The following secondary antibodies (antibody, dilution, vendor) were used: Goat anti-mouse IgG (H+L)-HRP conjugate, 1:2,000, Bio-Rad 170-6516; Goat anti-rabbit IgG (H+L)-HRP conjugate, 1:2,000, Bio-Rad 170-6515; Alexa Fluor 488 goat anti-rat IgG (H+L), 1:500, Invitrogen A11006; Alexa Fluor 594 goat anti-rabbit IgG (H+L), 1:500, Invitrogen A11012; Alexa Fluor 488 goat anti-mouse IgG (H+L), 1:500, Invitrogen A11029; and Alexa Fluor 488 goat anti-rabbit IgG (H+L), 1:500, A11008.

### Mice

#### Humanized I1061T NPC1 mice.

CRISPR/Cas9 gene editing was used to target exon 2 of the mouse *Npc1* gene. cDNA encoding human I1061T NPC1 (downstream of the human signal peptide) was inserted immediately after the mouse signal peptide coding sequence in *Npc1* exon 2 (as diagrammed in Figure 4) (Biocytogen). To create gene-targeted mice, the cDNA coding sequence for human I1061T NPC1 followed by a polyadenylation signal was inserted into a targeting vector containing mouse *Npc1* exon 2 and 5′ and 3′ homologous arms. sgRNAs selected by Biocytogen plus targeting vector and Cas9 RNA were microinjected into C57BL/6N zygotes, cultured, and then implanted into foster female mice. F_0_ mice were screened by PCR and those with 1 targeted *Npc1* allele sequenced through exon 2 to ensure insertion of human *NPC1* sequence at the correct position and in the proper orientation. Correctly targeted F_0_ mice were bred to C57BL/6 mice, and resulting F_1_ mice were analyzed by Southern blot to confirm single insertion of human NPC1 sequence (Supplemental Figure 6A). The established gene-targeted mouse line was maintained on the C57BL/6J background.

*Npc1^nih^* BALB/cJ mice were obtained from Jackson Laboratory (003092) and maintained on BALB/cJ background. F_1_ hybrid mice were generated by crossing heterozygous *Npc1^nih^* mice on BALB/cJ with humanized I1061T NPC1 on C57BL6/J background.

Mice were housed in a 12-hour dark/12-hour light cycle, in a facility with an ambient temperature ranging 20.6°C–23.9°C and a humidity between 30% and 70%. Serum liver function tests were performed by the University of Michigan In Vivo Animal Core.

#### Balance beam.

The balance beam consists of a 5 mm–wide square beam suspended at 50 cm. Mice were trained at 5 weeks of age to cross the beam and then tested every other week starting at 6 weeks. For testing, mice were first acclimated inside a dark plastic hut containing bedding for 5 minutes. Mice were then run 3 times across the beam with a 2-minute rest between runs, and the average time from 3 trials was calculated. Maximum time was set at 20 seconds, and falls were scored as 20 seconds.

#### Rotarod.

After acclimating to the testing room for 30 minutes, mice were gently placed on a moving (4 rpm) rotarod for 30 seconds. Then, over a period of 235 seconds, rotarod speed was increased to 40 rpm. Mice were trained at 6 weeks of age, then tested every other week starting at 7 weeks. Mice were run 3 times with a 5-minute rest between trials. The average time was calculated, with 240 seconds set as the maximum time. The trial ended when mice reached the maximum time, stopped walking for 2 revolutions, or dropped onto the paddle.

### Cell lines

#### Fibroblasts.

The following cell lines were obtained from the NIH National Institute of General Medical Sciences Human Genetic Cell Repository at the Coriell Institute for Medical Research: GM08399 (CTRL) and GM18453 (I1061T/I1061T). All fibroblasts were grown in Advanced MEM (Gibco 12492-013) + 10% FBS (R&D Systems, Bio-Techne, S11150) + 1× penicillin-streptomycin-glutamine (Gibco 10378016). Mouse fibroblasts were generated from animals as previously described (31). Fibroblasts from the humanized I1061T NPC1 mice were immortalized after a week of culture by transduction with lentiviral SV40 (Abm G203). Nontransduced cells were selected out by cell death after several passages to establish the cell line.

#### iPSCs.

NPC1-mutant iPSC lines were generated by the Genome Engineering & Stem Cell Center at Washington University in St. Louis (St. Louis, Missouri, USA) using CRISPR/Cas9 gene editing of the parental BJFF.6 iPSC line, as previously described (31). iPSCs were cultured on plates coated overnight with Geltrex (Thermo Fisher Scientific A1413302) diluted in DMEM/F12 (Gibco 11330-032). Every 24 hours, cells were fed with fresh StemFlex Basal Medium (Gibco A33493-01) with StemFlex Supplement. iPSCs were split by rinsing with DPBS, no calcium, no magnesium (DPBS-/-; Gibco 14190-144); incubating with Gentle Cell Dissociation Reagent (STEMCELL Technologies 100-0485) for approximately 5 minutes; and plating on new Geltrex-coated plates.

#### Generation of isogenic iNeurons.

The generation of I1061T NPC1 and R934L NPC1 iNeurons was previously described (31). The generation of P1007A NPC1 and R1186H NPC1 iNeurons was completed with the same protocol using the PiggyBac system (31). The guide RNAs and single-stranded oligo DNA nucleotides used to target and insert each indicated point mutation are shown in Supplemental Table 4. To insert NGN2 into iPSCs using PiggyBac transposase, Lipofectamine Stem Transfection Reagent (Thermo Fisher Scientific) was used to cotransfect expression vectors encoding 1) PiggyBac transposase (CMV PBase); and integrating vectors containing PiggyBac transpose ITR expressing 2) puromycin selectable marker (CAG-Puro); 3) CAG TetON-IRES-mCherry for estimating homogeneity of colonies; and 4) TRE-human NGN2, which enables NGN2 expression in the presence of doxycycline. To accomplish this, P1007A NPC1 and R1186H NPC1 iPSCs were plated on Matrigel-coated (Corning 354277) plates in the presence of 1 μM Y-26732 (Cayman Chemical 10005583) in StemFlex. On day 1, the media was changed to E8 (STEMCELL Technologies 05990), and cells were transfected using 10 μL Lipofectamine Stem Transfection Reagent diluted in 125 μL Opti-MEM (Gibco 31985062) containing 0.9 μg PBase, 0.7 μg mCherry, 0.7 μg hNGN2, and 0.2 μg Puromycin plasmids. On day 2, the media were changed to StemFlex. On day 3, cells were lifted into a single-cell dilution series using Accutase (STEMCELL Technologies 07922) and plated into plates containing StemFlex and Y-27632. On days 4–5, cells were fed with StemFlex. On day 6, antibiotic selection was performed with StemFlex containing 0.85 μg/mL puromycin (InvivoGen ant-pr-1). Selection was observed on day 7–8. On day 9–14, 10 mCherry-positive colonies were selected. From these 10, the most efficient clones were identified based on neuron differentiation.

#### iNeuron differentiation.

iPSCs were dissociated with Gentle Cell Dissociation Reagent (STEMCELL Technologies 100-0485) and plated on Geltrex-coated plates as with StemFlex media containing Y-27632 on day –1. On days 0 and 1, media were changed to StemFlex with 0.5 μg/mL doxycycline (MilliporeSigma D9891). On day 2, cells were washed with DPBS-/-, lifted with Accutase, and replated onto Geltrex-coated plates cultured with 3N media (4 mL penicillin/streptomycin, Gibco 15140; 250 mL DMEM/F12, Gibco 11330-032; Neurobasal, Gibco 21103-049; 125 μL 10 mg/mL insulin, MilliporeSigma I9278; 2.5 mL nonessential amino acids, Gibco 11140-050; 2.5 mL N2 supplement, Gibco 17502-048; 5 mL B27 supplement, Gibco 17504-044; 2 μL β-mercaptoethanol stock [12 M], MilliporeSigma M7522; and 2.5 mL Glutamax, Gibco 35050-061) containing 0.5 μg/mL doxycycline and Y-27632. On day 3, media were replaced with fresh 3N media with doxycycline. On day 4 and following, half-media changes were conducted daily.

#### iNeuron drug treatments.

Drug treatments were initiated on day 3 of iNeuron differentiation. Unless otherwise specified, all drug treatments were 48 hours. Drugs were used at the following concentrations: 10 μM abiraterone (Cayman Chemical Company 15148), 400 μM arimoclomol (synthesized at the University of Notre Dame), 0.2 μM CI-994 (synthesized at the University of Notre Dame), 1 μM mo56Hc (synthesized at Ochanomizu University and at the University of Notre Dame), 0.005 μM onalespib (synthesized at the University of Notre Dame), 10 μM quinestrol (Cayman Chemical Company 10006320), 0.1 μM U18666A (Cayman Chemical Company 10009085), 0.2 μM vorinostat (synthesized at the University of Notre Dame), 1 μM 25Hc (MilliporeSigma H1015), and 100 μM 4-phenylbutyric acid (MilliporeSigma P21005).

### Western blot

#### Cells.

After aspirating media, cells were washed with PBS, then removed with a cell scraper and centrifuged at 1,000*g* at 4°C for 5 minutes. The cell pellet was then resuspended in approximately 100 μL RIPA (Teknova R3792) containing cOmplete Mini protease inhibitor (Roche 46264500) and 0.625 mg/mL N-ethylmaleimide (Acros Organics 128-53-0). The RIPA cell suspension was sonicated until cells were completely dissociated. The resulting solution was then centrifuged at 3,000*g* for 5 minutes at 4°C, after which the supernatant, including suspended protein, was transferred. Protein concentrations were calculated using the DC-protein assay (Bio-Rad).

#### Tissue.

Tissues were collected from mice and flash-frozen in liquid nitrogen. Tissues were suspended in lysate buffer as above, then homogenized by a tissue homogenizer and sonicator. The resulting solution was centrifuged at 3,000*g* for 5 minutes at 4°C, and then the supernatant was transferred and quantified as above.

#### Both.

A total of 40 μg protein was loaded per well into NuPAGE 4%–12% gradient Bis-Tris gels (Invitrogen WG1401BX10). Gels were run for 1.5 hours (total protein) or 2 hours (Endo H) at 130 V in 1× NuPAGE MOPS SDS running buffer (Invitrogen NP0001). Gels were transferred to Immobilon-P 0.45 μm PVDF (Merck Millipore IPVH00010) membranes. Membranes were blocked for 1 hour at room temperature, then incubated in primary antibody overnight at 4^o^C. Membranes were incubated in secondary antibody at room temperature for 1 hour. Immunoreactivity was detected with SuperSignal West Pico PLUS Chemiluminescent Substrate (Thermo Fisher Scientific 34577) via an iBright FL1500 imaging system (Invitrogen A44241). Quantification of bands within the linear exposure range was performed using Fiji (NIH), and band intensity was normalized to the indicated loading control. If brightness and contrast were modified, adjustments were performed equally to the entire image and controls after quantification.

### Endo H assay

Reactions of 20 μL containing 40 μg protein and 2 μL glycoprotein denaturing buffer were incubated at 37°C for 15 minutes. After incubation, the samples received 4 μL 10× glycobuffer 3, 12 μL water, and 4 μL Endo H enzyme (New England BioLabs P0702L). Reactions were incubated at 37°C for 3 hours. Reactions were terminated with 12 μL 4× NuPAGE LDS sample buffer (Invitrogen 2152677) mixed with 1 M DTT (MilliporeSigma D0632) for Western blot analysis.

### qPCR

#### RNA collection.

RNA was isolated from adherent cells using TRIzol Reagent (Ambion 15596018), following manufacturer’s instructions.

#### qPCR.

RNA was converted to cDNA using the High Capacity Reverse Transcription Kit (Applied Biosystems 4368814). qPCR was conducted in technical triplicates using 10 ng cDNA and TaqMan probes (Thermo Fisher Scientific) for 18S (4310893), Syn1 (Hs01018066), Map2 (Hs00258900), and MapT (Hs00902194). Intercalating dye qPCR was performed using SYBR Green reagents (Roche 4913850001). Primers were designed to span exon junctions using IDT PrimerQuest. For humanized I1061T NPC1: sense, AGCCGAACTGAGAGCTGTA; antisense, CCACACTCTCCATACCAAACAC. For mouse Cpsf2: sense, CCACCAGTGAAACGCATATCTA; antisense, ACGCCATCTATCCAAGCTAAC. qRT-PCR was performed using Applied Biosystems 7900HT Sequence Detection System, and relative expression was calculated by the 2^–ΔΔCT^ method using SDS software.

### Fluorescence staining

#### Immunofluorescence staining.

Staining of iNeurons and fibroblasts was performed as previously described (31) with slight modifications. All cells were washed twice with DPBS+/+ and fixed with 4% paraformaldehyde (PFA) (Electron Microscopy Sciences 15710) for 20 minutes. After washing twice again, cells were permeabilized using 0.1% Triton X-100 (MilliporeSigma) for 3 minutes, then blocked for 40 minutes in block solution (10% NGS, 1% BSA, PBS). Cells were incubated overnight at 4°C with appropriate primary antibodies diluted in block solution. The following day cells were washed twice in DPBS+/+, then labeled with appropriate secondary antibodies for 1 hour at room temperature. Cells were washed twice in DPBS+/+ and mounted with VECTASHIELD Antifade Mounting Medium with DAPI (Vector Laboratories H-1200).

For tissue staining, mice were perfused with saline and 4% PFA. Then, tissue was removed and postfixed in 4% PFA overnight at 4°C. Brains were bisected; one hemisphere was processed for paraffin embedding, and the other was incubated in 30% sucrose for 48 hours at 4°C and frozen in O.C.T. (Thermo Fisher Scientific 23730571). Paraffin-embedded tissues were processed and stained with H&E by the University of Michigan Tissue and Molecular Pathology Shared Resource. Frozen sections were prepared at 10 μm on a cryostat. Tissues were permeabilized in 0.1% Triton X-100, 10% NGS, and 1% BSA in PBS for 30 minutes. Slides were then placed in blocking solution (10% NGS, 1% BSA in PBS) before incubating in primary antibody diluted in blocking solution overnight at 4°C. Slides were washed 3 times in PBS and incubated for 1 hour with secondary antibody diluted in blocking solution. Slides were mounted with VECTASHIELD + DAPI (Vector Laboratories H-1200).

#### Filipin staining.

Fibroblasts were stained with filipin as previously described (31), with the following modification: after removal of filipin labeling solution, nuclei were stained with Sytox Green (Thermo Fisher Scientific S7020) at 125 nM for 15 minutes. Cells were then washed 3 times in PBS and mounted with ProLong Gold (Thermo Fisher Scientific P36030). Images were quantified by multiplying intensity of positive filipin puncta by area occupied, divided by nuclei number. A total of 8 images per well were averaged for each data point.

For tissues, slides were permeabilized in 4% PFA + 0.1% Triton X-100 for 30 minutes, then incubated in 1.5 mg/mL glycine for 10 minutes. Slides were blocked and stained with primary antibody as above. Following washes and secondary antibody incubation, slides were incubated with filipin staining solution (1 mL FBS + 9 mL PBS + 40 μL filipin in DMSO) for 2 hours at room temperature. Slides were washed and mounted with VECTASHIELD (Vector Laboratories H-1000).

### Microscopy

#### Epifluorescence microscopy.

Images were captured on an epifluorescence microscope using ZEISS imaging software. Cells were focused using DAPI, and sequential tiling was used to capture sets of 4 images at 20× original magnification. For every experimental and control group, 6 tiling sets were captured with approximately 183–308 cells/set. Images were quantified using NIH ImageJ, and the percentage of Map2-positive cells was reported. H&E-stained sections were imaged using bright-field settings. Brightness and contrast were applied equally across the entire image and to controls using Adobe Photoshop.

#### Confocal microscopy.

Images were captured on a Nikon X1 Yokogawa Spinning Disk Confocal microscope. Cells were focused by nuclear stain, and images were captured at indicated magnification. Images were quantified using a CellProfiler pipeline. Brightness and contrast were applied equally across the entire image and to controls using Photoshop.

### Nano-differential scanning fluorimetry

#### Expression and purification of recombinant NPC1 protein.

The full-length cDNA of WT human NPC1 was cloned into a pEG-BacMam vector with a C-terminal Strep tag. The protein was expressed using baculovirus-mediated transduction of mammalian HEK293S GnTI^−^ cells (American Type Culture Collection; ATCC). Baculovirus was generated in Sf9 cells (ATCC), and P2 virus was used to infect HEK293S GnTI^−^ cells at 37°C. At 8 hours after infection, sodium butyrate at a final concentration of 10 mM was added to the culture. After further incubation for 36 hours at 37°C, 800 mL cells were harvested and resuspended in 20 mL Buffer A (20 mM HEPES [pH 7.5] and 150 mM NaCl) supplemented with 1 mM PMSF and leupeptin (10 μg/mL) and then homogenized by sonication. The lysate was cleared by centrifugation at 4,000 rpm for 5 minutes at 4°C. The resulting supernatant was supplemented with 1% n-Dodecyl-β-d-Maltopyranoside (DDM; D310, Anatrace) and incubated for 1 hour at 4°C. Insoluble components were removed by centrifugation at 18,000 rpm, 4°C, for 30 minutes, and the clarified lysate was then loaded onto a disposable gravity column (Bio-Rad) containing 1 mL dry Strep-TactinXT 4Flow resin (IBA). The column was washed with 20 mL of Buffer B (20 mM HEPES [pH 7.5], 150 mM NaCl, 0.02% DDM). The resin was then incubated with 1 mL of 5 mg/mL nanodisc scaffold peptide (NSPr) solution in Buffer A at 4°C for 30 minutes before being washed with 10 mL of 1 mg/mL peptide NSPr solution in Buffer A and then 20 mL with Buffer A alone. Retained NPC1 protein was eluted with 10 mL of Buffer A supplemented with 50 mM biotin. Final purification was achieved by gel filtration (Superose 6 Increase 10/300, Cytiva) in Buffer A. The homogeneity of the final preparation was evaluated by SDS-PAGE (4%–12% gradient) with Coomassie stain visualization of 0.7 μg NPC1 per well (see Supplemental Figure 7).

#### Nano-differential scanning fluorimetry.

Purified recombinant WT NPC1 protein formulated in peptidiscs (gift of Xiaochun Li and Tao Long, UT Southwestern Medical Center, Dallas, Texas, USA) was diluted to 1.4 μM in 20 mM HEPES, 150 mM NaCl, pH 7.5, before addition of 1% DMSO, 50 μM mo56Hc in 1% DMSO, or 50 μM 25Hc in 1% DMSO. All treated samples were incubated on ice for 30 minutes before loading 10 μL of each sample into Prometheus Standard capillaries for analysis using the Prometheus Panta instrument (Nanotemper). The intrinsic tryptophan fluorescence of each protein solution was measured (excitation = 280 nm, emission = 330 nm and 350 nm) as the temperature of the chamber was increased at 3.5°C/min from 35°C to 95°C. The first derivatives of the emission signals at 330 nm were determined, and those data are plotted to clearly define the melting temperature of each thermal transition.

### Lipidomics

#### Lipid extraction for liquid chromatography–MS.

Brain and liver tissue samples were homogenized by probe sonication in 1× PBS buffer containing phosphatase inhibitors [1 mM NaF, 1 mM β-glycerophosphate, 1 mM PMSF, 1 mM Na_3_VO_3_, 2.5 mM Na_4_(PO_4_)_2_] and the protease inhibitor mixture (MilliporeSigma, S8820). Following the tissue homogenization, protein concentration was measured using the Pierce bicinchoninic acid assay (Thermo Fisher Scientific 23225). Organic extraction of lipids from the tissue lysates was performed via Folch lipid extraction method (50). Briefly, 100 μg protein equivalent lysate was taken, and the volume was adjusted to 40 μL using the 1× PBS lysis buffer. EquiSPLASH LIPIDOMIX quantitative standard mixture (Avanti Lipids 330731) was added as internal lipid standards, and lipids were extracted using 800 μL of 2:1 (v/v) chloroform/methanol mixture. The organic layer was dried under vacuum and resuspended in 100 μL of 1:3 (v/v) chloroform/methanol mixture for liquid chromatography–MS analysis.

#### Liquid chromatography–MS analysis.

Lipid separation was performed using an Agilent 1290 Infinity II UHPLC system equipped with an Agilent Poroshell 120 EC-C18 chromatography column (2.1 × 100 mm, 2.7 μm). Mobile phases consisted of solvent (A) 90:10 (v/v) water/methanol with 10 mM ammonium acetate and solvent (B) 20:30:50 (v/v) acetonitrile/methanol/isopropanol with 10 mM ammonium acetate. The column was maintained at 50°C and operated at a flow rate of 0.3 mL/min. From each sample, 1.5 μg protein equivalent was injected for positive-ionization mode, and 2 μg was injected for negative-ionization mode. Data acquisition was done using an Agilent 6550 quadrupole time-of-flight mass spectrometer scanning *m/z* range 200–1,700 in MS-only mode. A pooled lipid sample for each liver and brain was made by mixing equal amounts of all biological samples and analyzed using the iterative MS/MS mode with a fixed collision energy of 25 eV.

#### Data analysis.

Lipid identification was performed using the Agilent Lipid Annotator software, and a library of identified lipids was created from the pooled sample. Peak integration of the extracted ion chromatogram for each *m/z* value was done in Agilent Profinder software. The library was used further to filter the data for relative quantification and statistical analysis.

### Statistics

Statistical tests were performed in GraphPad Prism (v9.4.1). Single comparisons were analyzed by unpaired 2-tailed *t* test. Multiple comparisons were analyzed by 1-way ANOVA with Tukey’s post hoc multiple comparisons test. Groups with multiple variables were analyzed by 2-way ANOVA with Bonferroni’s post hoc comparisons test. Normal distribution of data was assumed. Differences in survival were assessed by log-rank test. *P* values of less than or equal to 0.05 were considered significant.

### Study approval

All procedures involving mice were approved by the University of Michigan Committee on Use and Care of Animals (PRO00010017) and conducted in accordance with institutional and federal guidelines.

### Data availability

Data are available in the Supporting Data Values XLS file.

## Author contributions

MLS, RAF, SMC, and APL conceived the study. SMC, MLS, RAF, BSJB, and APL developed methodology. RDA, ABC, KJS, MZ, KCP, VM, DTN, and BLA performed investigation. KO, PH, and OW contributed unique reagents. MLS, SMC, RAF, PH, BSJB, and APL acquired funding. MLS, APL, SMC, RAF, and BSJB performed project administration. MLS, APL, SMC, RAF, and BSJB supervised. RDA, ABC, KJS, MLS, and APL wrote the manuscript. RDA, ABC, KJS, MZ, KCP, VM, DTN, BLA, PH, OW, KO, QL, RAF, BSJB, MLS, SMC, and APL reviewed and edited the manuscript.

## Supplementary Material

Supplemental data

Unedited blot and gel images

Supplemental table 3

Supporting data values

## Version 1 08/29/2024

In-Press Preview

## Version 2 10/22/2024

Electronic publication

## Version 3 10/25/2024

Kylie J. Schach's initials were corrected in the authorship note
