# Supplementary material for: Mutant induced neurons and humanized mice enable identification of Niemann-Pick type C1 proteostatic therapies
Source: JCI Insight. 2024 Oct 22;9(20):e179525. doi: 10.1172/jci.insight.179525 (PMC11530122; doi:10.1172/jci.insight.179525)
Supplement: Supplemental data [file jciinsight-9-179525-s321.pdf]

Supplementary Methods and Data

Mutant induced neurons and humanized mice enable identification of Niemann-Pick C1 proteostatic therapies

SUPPLEMENTARY METHODS

Cell viability: To determine non-toxic doses of the various drugs employed in this study, iNeurons were treated with vehicle or varying drug concentrations in triplicate for 48 hours in a 96-well plate. Cell viability was determined using the CyQuant XTT Cell Viability Assay (Thermo Fisher X12223) following the manufacturer's instructions. Absorbance was read after approximately 2 hours of incubation.

Immunofluorescent live cell imaging: iNeurons were incubated with 50nM LysoTracker Deep Red (Thermo Fisher L12492) at 37°C for 30 minutes. After 10 minutes of LysoTracker staining, 2 drops of NucBlue Live ReadyProbes Reagent (Thermo Fisher R37605) per mL of culture media was added to plates. At 30 minutes, media with staining reagents was replaced phenol red-free DMEM/F12 (Thermo Fisher 21041025) with 0.5 μg/mL doxycycline. Cells were imaged on a Nikon X1 Yokogawa Spinning Disk Confocal microscope with stage-top incubator. Images were quantified using a CellProfiler pipeline. Brightness and contrast were applied equally across the entire image and to controls using Photoshop.

Microscale Thermophoresis (MST) assay. NPC1 protein was labeled with Mal-NT650 dye for 30 minutes at room temperature, then purified on a PD MiniTrap column, eluted, and centrifuged at 15,000 rpm for 5 minutes at 8° C. Labeled, resuspended NPC1 protein was mixed with a dilution series of mo56Hc and incubated at room temperature for 30 minutes. The solution was loaded into Monolith NT.115 Series MST Premium Coated Capillaries and analyzed on a Monolith NT.115 (NanoTemper Technologies GmbH).

SUPPLEMENTARY REFERENCES

1. Long T, Qi X, Hassan A, Liang Q, De Brabander JK, and Li X. Structural basis for itraconazole-mediated NPC1 inhibition. *Nat Commun.* 2020;11(1):152.

SUPPLEMENTARY TABLES

STR	Parental WT Line	P1007A
AMELX	X, Y	X, Y
D21S11	29	29
D19S433	16	16
D13S317	8, 9	8, 9
TPOX	10, 11	10, 11
D7S820	11, 12	11, 12
D5S818	12	12
CSF1PO	10, 12	10, 12
D16S539	9, 13	9, 13
D3S1358	14, 16	14, 16
TH01	7, 8	7, 8
D8S1179	9, 11	9, 11
VWA	16, 18	16, 18
FGA	22, 23	22, 23
D2S1338	16, 20	16, 20
D18S51_#1	17, 19	17, 19

Supplementary Table 1. Short tandem repeat analysis of P1007A iPSC line.

STR	Parental Line	R1186H
AMELX	X, Y	X, Y
PentaE	7, 17	7, 17
D21S11	29	29
D19S433	16	16
D13S317	8, 9	8, 9
TPOX	10, 11	10, 11
D7S820	11, 12	11, 12
D5S818	12	12
CSF1PO	10, 12	10, 12
D16S539	9, 13	9, 13
D3S1358	14, 16	14, 16
TH01	7, 8	7, 8
D8S1179	9, 11	9, 11
VWA	16, 18	16, 18
FGA	22, 23	22, 23
D2S1338	16, 20	16, 20
D18S51_#1	17, 19	17, 19
PentaD	12, 13	12, 13

Supplementary Table 2. Short tandem repeat analysis of R1186H iPSC line.

Target	gRNA	donor/ssODN
NPC1-R1186H	cctcttccgcgcgctccacgNGG	cggcgtggccctgctcagggtactcacggagctgcccatgtgggcaagtgcctcttccgcATGctccacgcggctgcctttcatgctcaccgtgaacgctctggttatgtggctgcagaactccacg
NPC1-P1007A	tggggttAGGgttatccgaaNGG	gttgcagtggatgcttatctgcaatggcagcagcacttaccctttgccacacttggggttAGCgttatcGgaaaggaacatgggcaggaatctcatgaagtctcccccctgaggcctctgtttgccttcc
NPC1-R934L	gtcctctctagtaccCGAatNGG	aaactaaagacttcctccctgtggagcaggtcagtaaccctgtctgtcctctctagtaccCTTataggcttcgccccctcgtcctggatcgacgattatttcgactgggtgaagccacagtcg
NPC1-I1061T	gacattactggctAtaagtcNGG	taaggaaatactcggtaggcactgccgttaatgcccatggtttcggtgacattactggctGtaagtcgggctttcttcagagcgtcaataaagtcagcagaggtctgcagcacggtgtggtagg

Supplementary Table 4. Guide RNAs and single stranded oligo DNA nucleotides used in gene editing.

SUPPLEMENTARY FIGURES

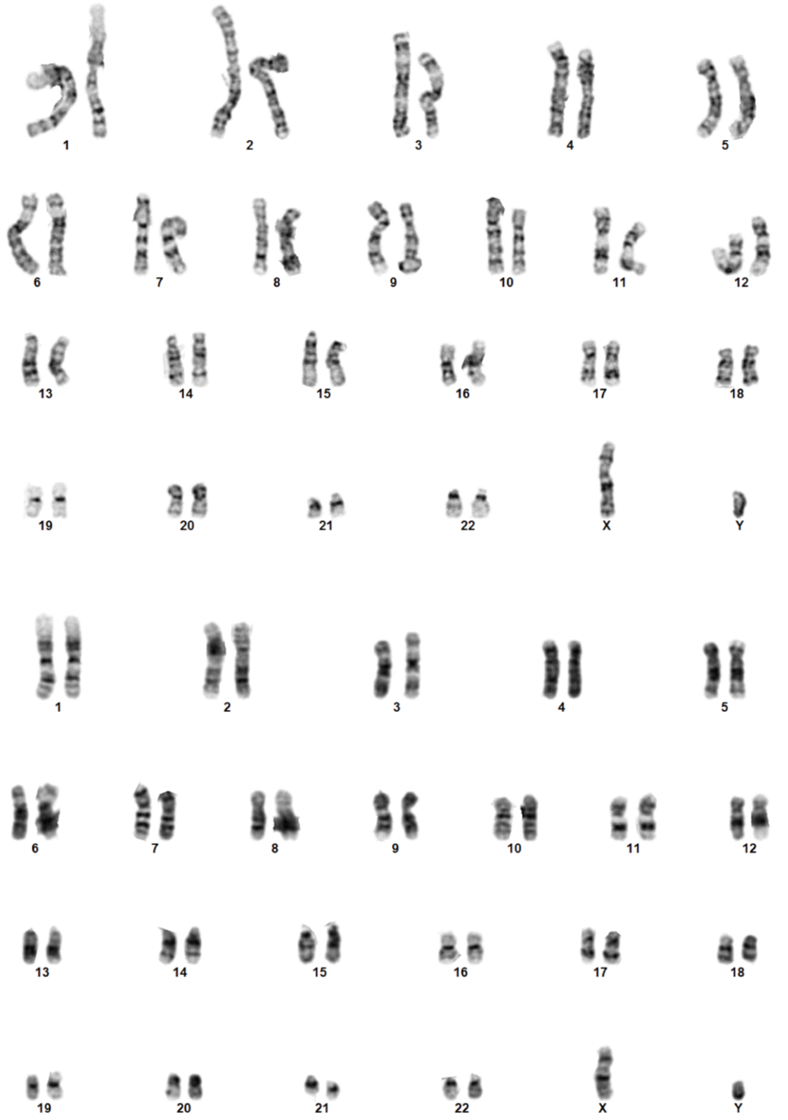

P1007A

R1186H

Supplementary Figure 1. CRISPR edited iPSCs have a normal karyotype. Karyotypes of P1007A and R1186H iPSCs were analyzed by the Cytogenetics Core at Washington University Saint Louis.

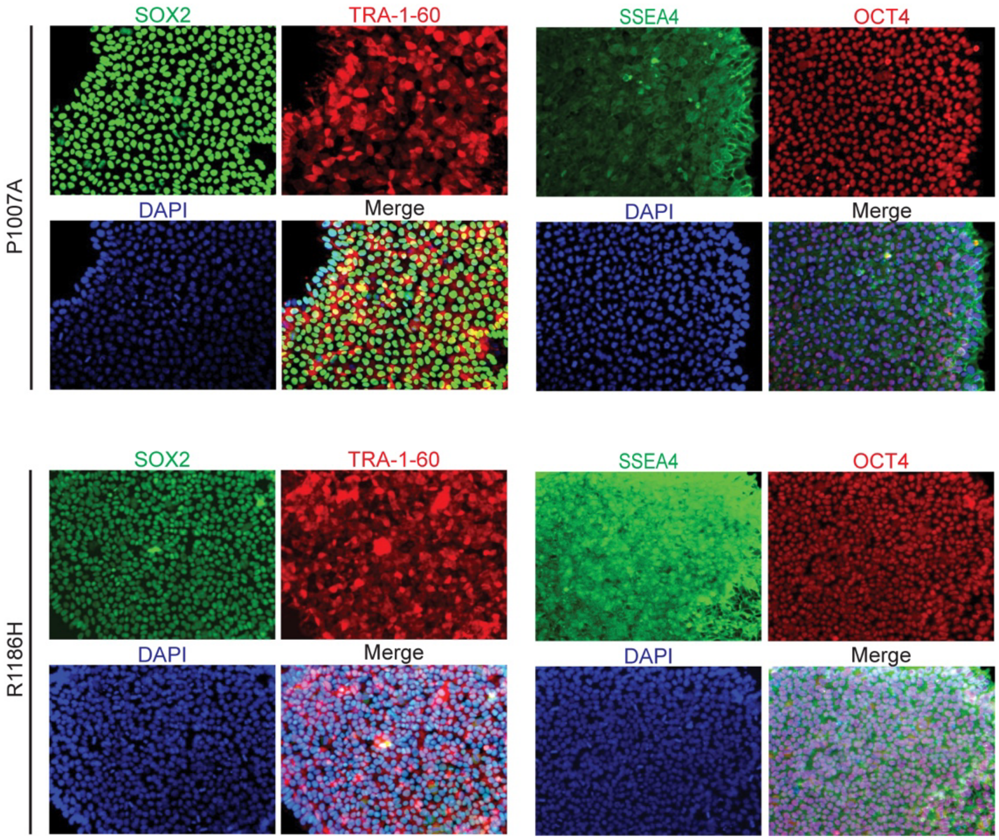

Supplementary Figure 2. Pluripotency analysis of iPSC lines. P1007A or R1186H iPSCs were stained with the pluripotency markers SOX2 (green), TRA-1-60 (red), SSEA4 (green), OCT4 (red). DNA was stained with DAPI (blue).

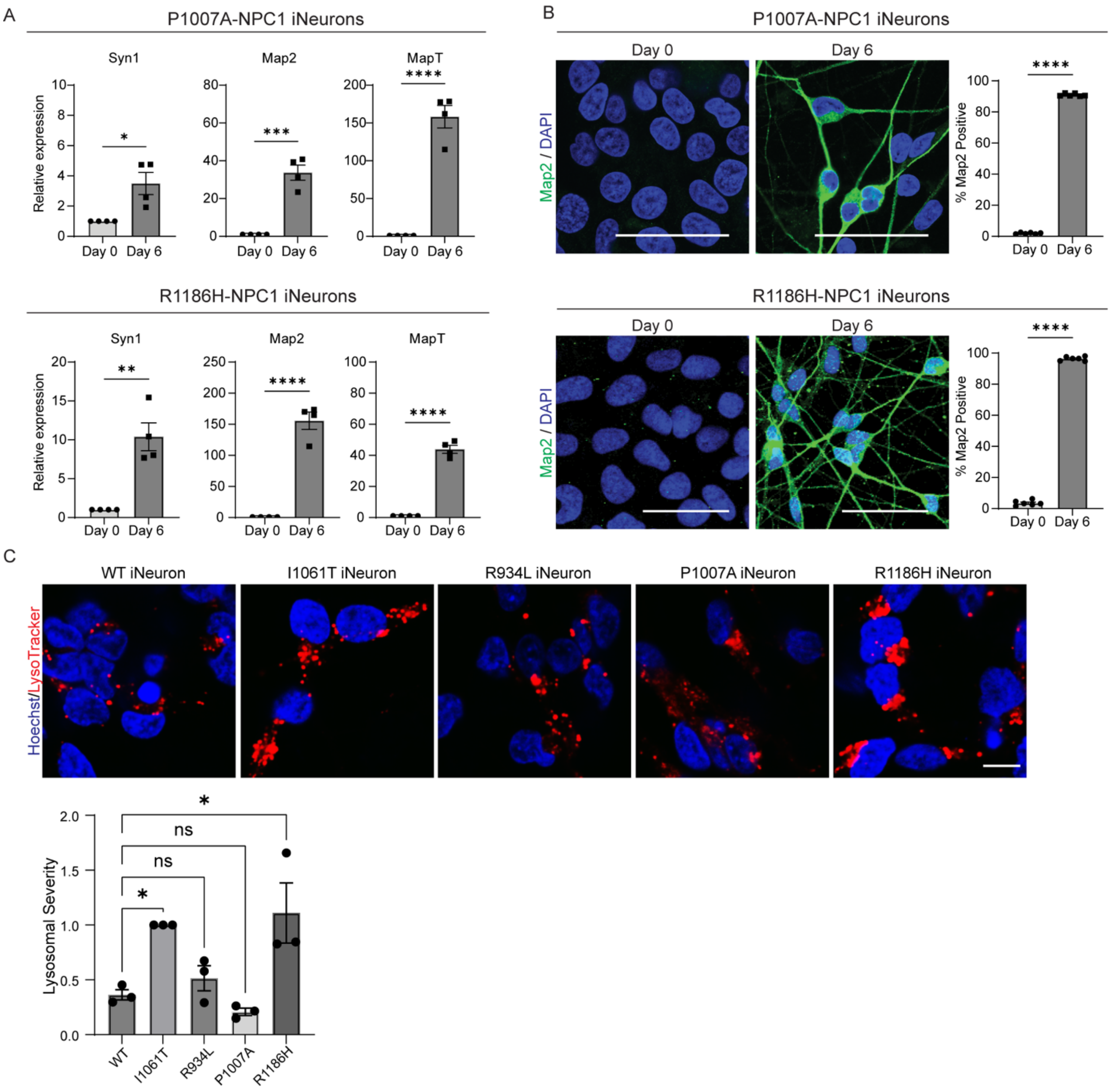

Supplementary Figure 3. Characterization of iPSC-derived iNeurons. (A) Differentiation of P1007A and R1186H NPC1 iPSCs to iNeurons was confirmed by qPCR using probes for SYN1, MAP2, and MAPT before (day 0) and after (day 6) differentiation. Day 0 or 6 indicates the duration of doxycycline treatment. **(B)** The same experimental groups were analyzed via staining for Map2 (green) and DAPI (blue). Percentage of Map2 positive somas with projections

quantified at right. Scale bars (white) are 50 µm. **(C)** WT or mutant iNeurons were differentiated and stained with LysoTracker (red) and Hoechst (blue). Severity of lysosomal staining was measured by multiplying LysoTracker staining intensity by area of image covered, then dividing by nuclei count. Images quantified below. Scale bar (white) = 10µm.

Data are mean ± s.e.m. from indicated number of independent experiments. **P* ≤0.05, ***P* ≤0.01, ****P* ≤ 0.001, *****P* ≤ 0.0001 by **(A, B)** two-tailed t-test or **(C)** one-way ANOVA. **(A)** P1007A (Syn1, Map2, MapT): n=4, 4, 4; R1186H: n=4, 4, 4; **(B)** P1007A, R1186H: n=6, 6; **(C)** n=3.

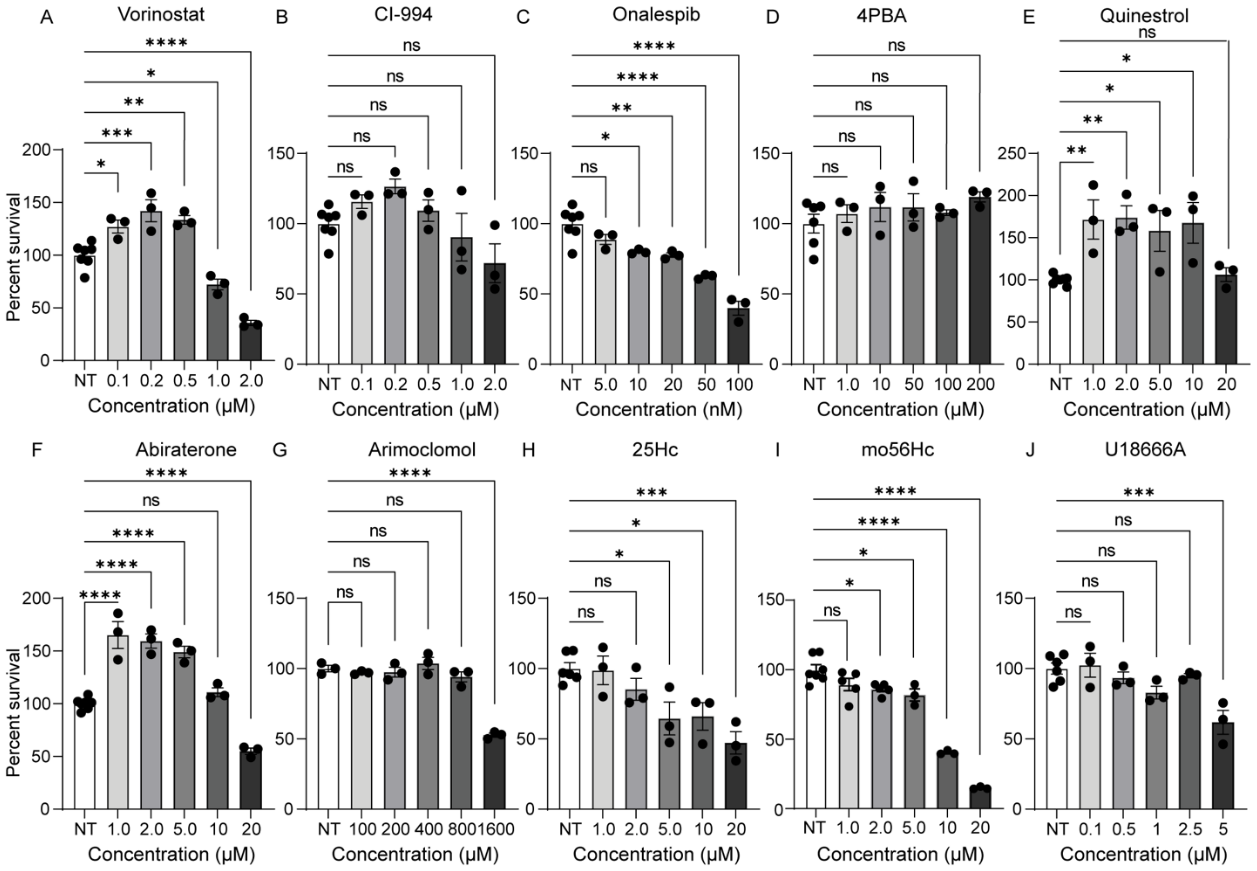

Supplementary Figure 4. Toxicity of proteostatic modulators in I1061T iNeurons. I1061T NPC1 iNeurons were treated for 48 hours beginning on day 3 of differentiation with the indicated concentrations of vorinostat, CI-994, onalespib, 4-phenylbutyric acid (4PBA), quinestrol, abiraterone, arimoclomol, u18666a, 25-hydroxycholesterol (25Hc), or mo56Hc. Cell viability was quantified using the CyQuant XTT Cell Viability Assay. Data are mean ± s.e.m. from indicated number of independent experiments. ns, not significant, **P* ≤0.05, ***P* ≤0.01, ****P* ≤ 0.001, *****P* ≤ 0.0001 by one-way ANOVA with Tukey's posthoc test. For drugs where the highest non-toxic dose was not selected in the study, morphologic changes were observed which led to the selection of a lower dose. **(A-C, E-F)** n=7, 3, 3, 3, 3, 3; **(D, H,J)** n=6, 3, 3, 3, 3, 3; **(G)** n=3, 3, 3, 3, 3, 3; **(I)** n=7, 5, 5, 3, 3, 3.

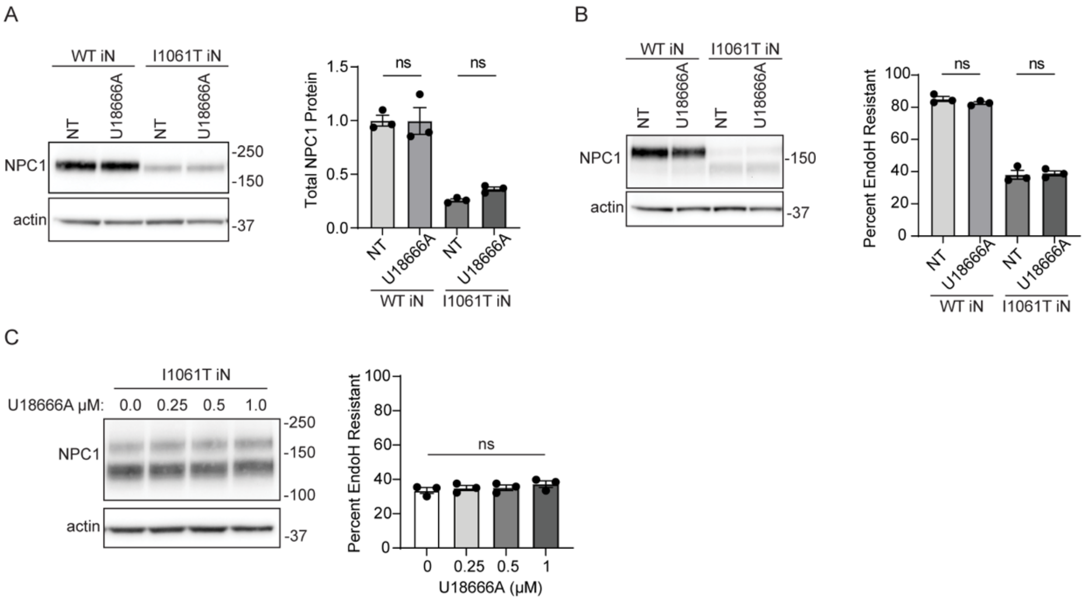

Supplementary Figure 5. U18666A does not alter total or EndoH resistant WT or I1061T NPC1. **(A-B)** WT or I1061T NPC1 iNeurons were treated for 48 hours beginning on day 3 of differentiation with 0.1 µM U18666A. Lysates were **(A)** analyzed via western blot for total NPC1 protein (quantified at right) or **(B)** digested with Endo H and analyzed via western blot for percent Endo H resistant NPC1 (quantified at right). **(C)** I1061T NPC1 iNeurons were treated for 24 hours with indicated concentrations of U18666A. Lysates were digested with Endo H and analyzed via western blot for percent Endo H resistant NPC1 (quantified at right). Data are mean ± s.e.m. from indicated number of independent experiments. ns, not significant by one-way ANOVA with Tukey's post-hoc test. **(A-C)** n=3.

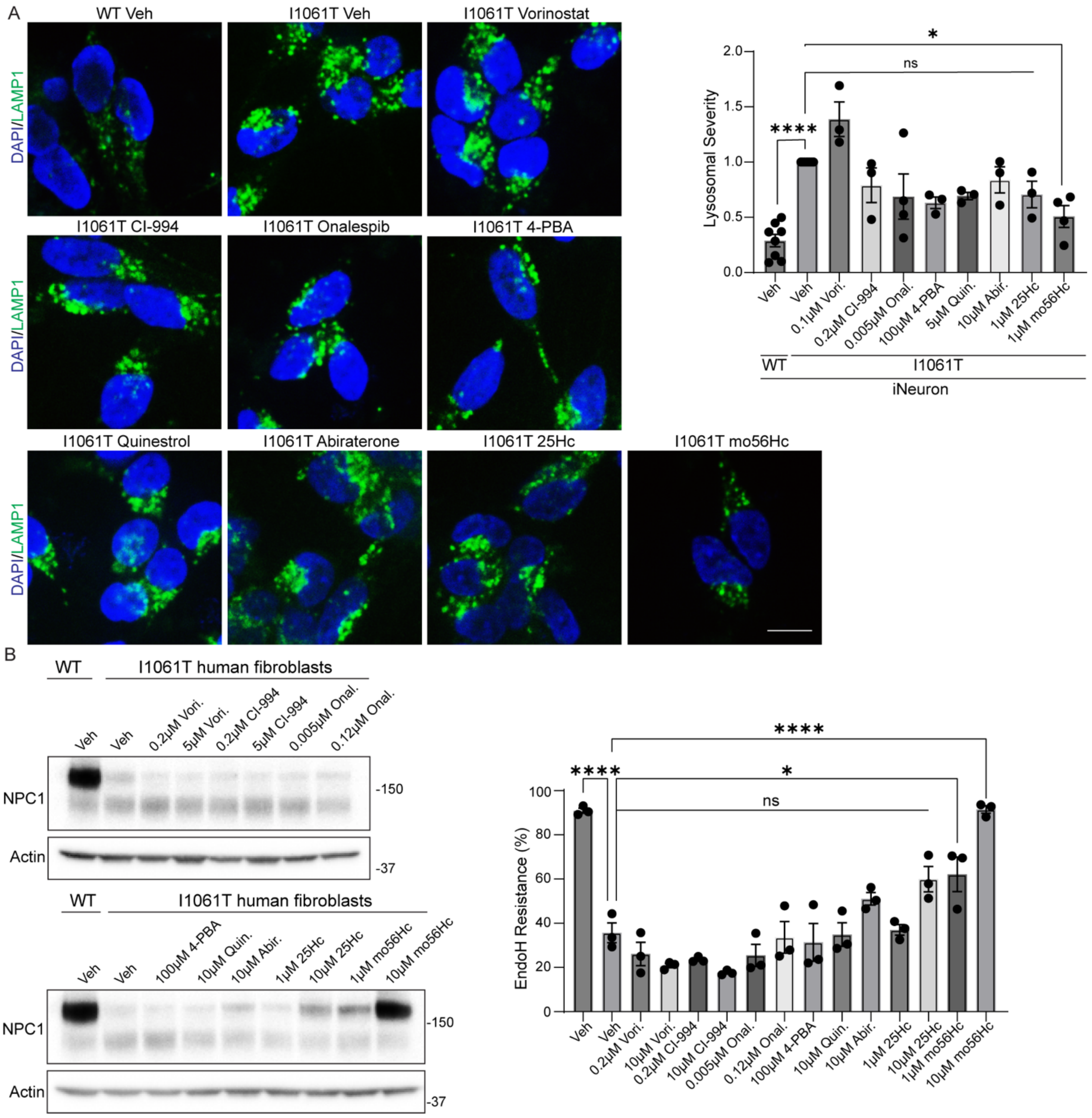

Supplementary Figure 6. Effects of proteostatic modulators on cellular phenotypes. (A) WT or I1061T NPC1 iNeurons were differentiated and treated with proteostatic modulators at indicated doses for 48h. Cells were stained with LAMP1 to label lysosomes and DAPI to label nuclei. Severity of lysosomal staining was measured by multiplying LAMP1 staining intensity by area of image covered, then dividing by nuclei count. Images quantified at right. Scale bar (white) = 10μm. **(B)** WT or I1061T NPC1 homozygous human fibroblasts were treated with

proteostatic modulators at indicated doses for 48h. Lysates were digested with Endo H and analyzed via western blot for percent Endo H resistant NPC1 (quantified at right). Data are mean ± s.e.m. from indicated number of independent experiments. *$P \leq 0.05$, ****$P \leq 0.0001$ by **(A, B)** one-way ANOVA. **(A)** n=8, 8, 3, 3, 4, 3, 3, 3, 3, 4; **(B)** n=3.

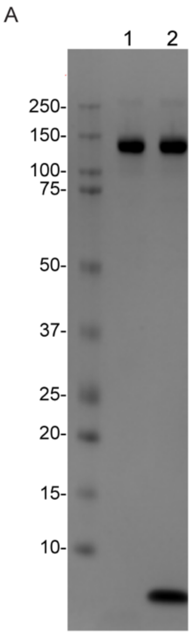

Supplementary Figure 7. NPC1 protein preparations. (A) Purified wild-type NPC1 proteins run at 0.7 μg per lane on a 4-12% gradient SDS-PAGE gel to demonstrate the homogeneity of the preparations. The gel was stained with Coomassie blue. Lane 1: NPC1 in 20mM Hepes pH7.5, 150mM NaCl and 0.02% GDN, Lane 2: NPC1 Peptidisc formulation in 20mM Hepes pH7.5, 150mM NaCl.

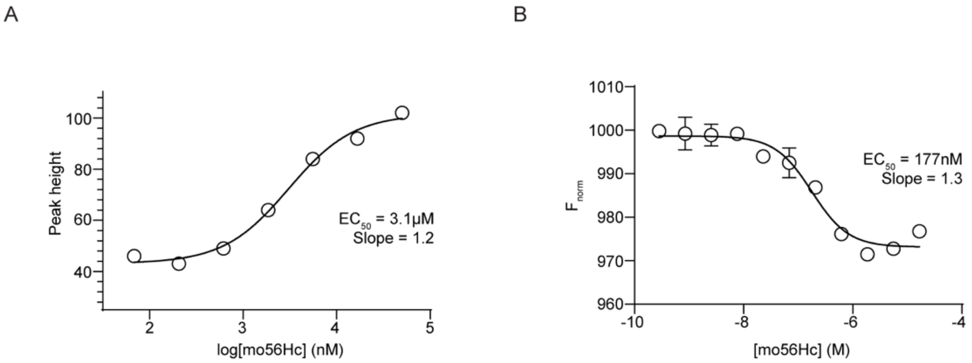

Supplementary Figure 8. Concentration dependence of mo56HC binding and stabilization of isolated NPC1 protein. **(A)** Impact of mo56HC on NPC1 thermal stability using the Nanoscale Differential Scanning Fluorescence (nanoDSF) assay. Stabilization was monitored via the height of the thermal transition peak in the first derivative transformation of the 330 nm fluorescent signal (reflecting an increase in the slope of the thermal transition). (**B)** Impact of mo56HC on the thermophoresis of isolated NPC1 protein using the Microscale Thermophoresis (MST) assay. Binding was monitored by the change in fluorescence in the area of laser-induced heat induction as the fluorescently-labelled NPC1 protein diffused across a thermal gradient. Ligand binding can change the rate of protein thermophoresis, as is evident in mo56HC binding to NPC1.

A

Southern Blot Strategy			
Restriction Enzyme	Probe	WT	Targeted
BclI	5' Probe	--	7.6kb
BspHI	3' Probe	11.4kb	7.9kb

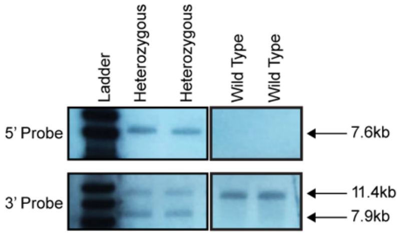

B

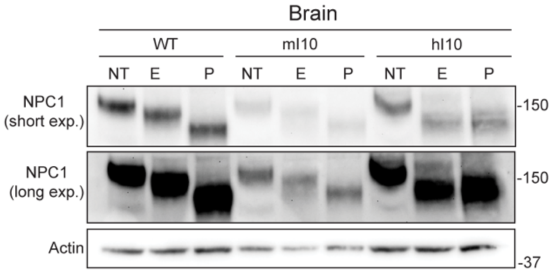

Supplementary Figure 9. Generation of humanized I1061T NPC1 mice.

(A) Southern blot analysis of genomic DNA from wild type and heterozygous humanized I1061T NPC1 mice. DNA was digested with indicated restriction enzymes and hybridized to 5' and 3' probes as indicated in Fig. 4A. **(B)** Brain lysates from WT, mI10, and hI10 mice were digested with Endo H and subject to western blot.

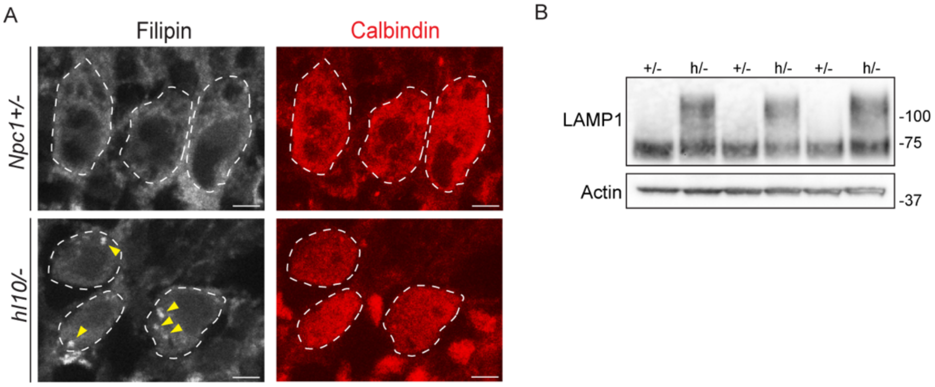

Supplementary Figure 10. Neuropathology of humanized I1061T NPC1 mice.

(A) Purkinje neurons in the cerebellum of 16-week-old mice were stained for calbindin (red) and unesterified cholesterol (filipin puncta, yellow arrowheads). Scale bar = 10 μm. **(B)** Cerebellar lysates from 52-week-old *Npc1*+/- (+/-) and *hI10/-* (h/-) mice were probed for LAMP1.

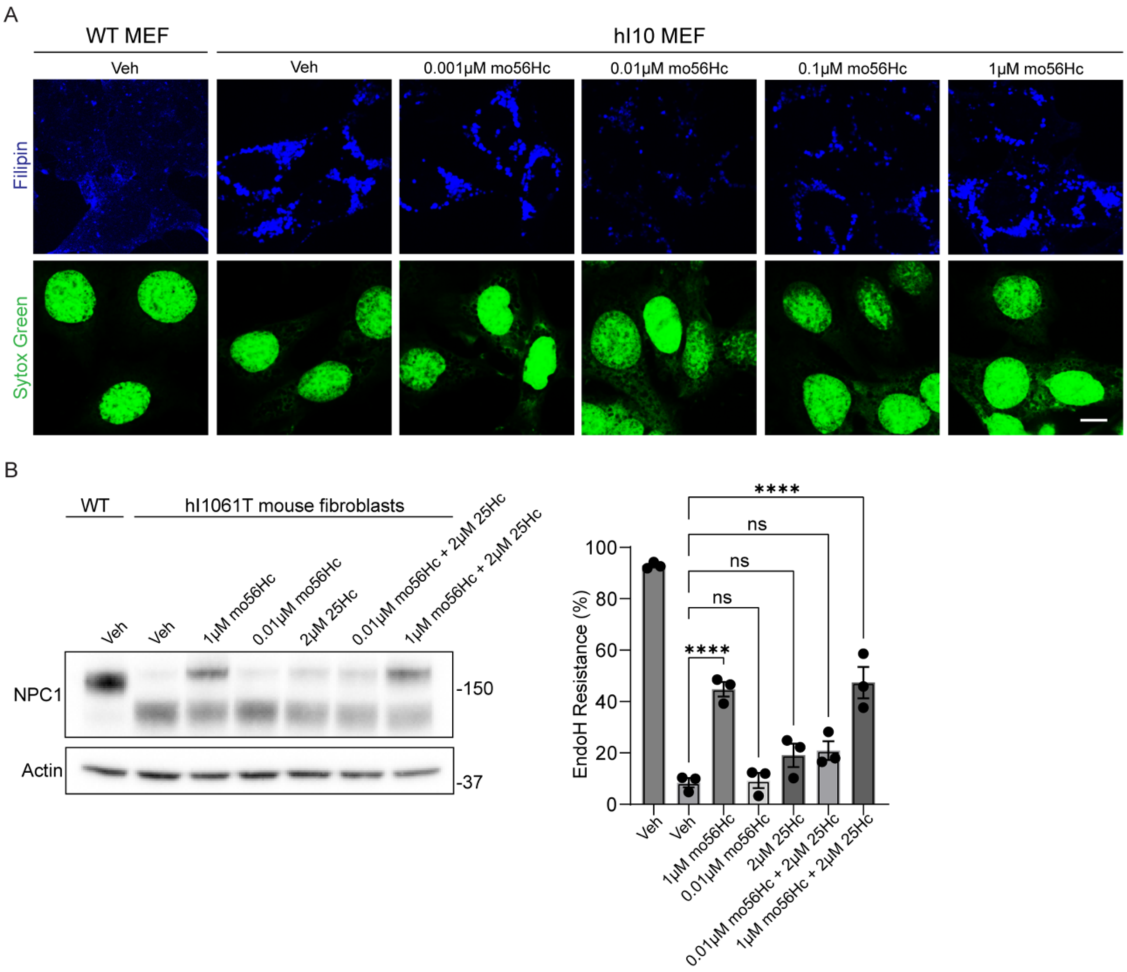

Supplementary Figure 11. mo56Hc corrects cholesterol storage in a dose-dependent manner. (A) Fibroblasts from WT or hI10 mice were treated with vehicle or varying doses of mo56Hc for 48h. Cholesterol was labeled with filipin and nuclei were labeled with Sytox Green. Scale bar = 10µm. **(B)** Fibroblasts from WT or hI10 mice were treated with vehicle, mo56Hc at varying doses, 25Hc, or a combination of the two for 48h. Lysates were digested with Endo H and analyzed via western blot for percent Endo H resistant NPC1 (quantified at right). Data are mean ± s.e.m. from indicated number of independent experiments. ****$P \leq 0.0001$ by **(B)** one-way ANOVA. **(B)** n=3.

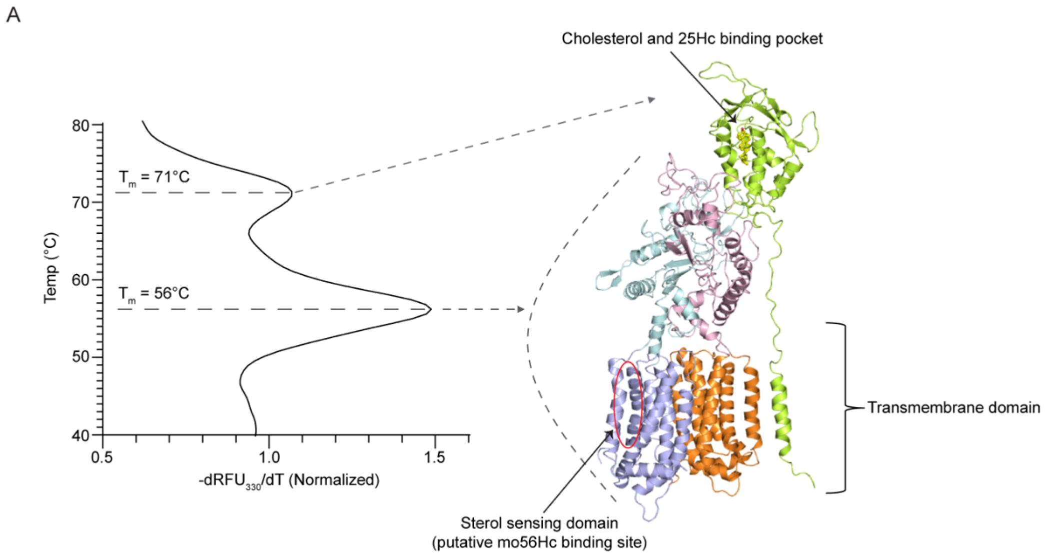

Supplementary Figure 12. Schematic illustrating proposed relationships between NPC1 thermal transitions and the protein's structure. NPC1 structure is from (1).
