# Supplementary material for: Mutant induced neurons and humanized mice enable identification of Niemann-Pick type C1 proteostatic therapies
Source: JCI Insight. 2024 Oct 22;9(20):e179525. doi: 10.1172/jci.insight.179525 (PMC11530122; doi:10.1172/jci.insight.179525)
Supplement: Unedited blot and gel images [file jciinsight-9-179525-s322.pdf]

Uncropped blots and images

Mutant induced neurons and humanized mice enable identification of Niemann-Pick C1 proteostatic therapies

Fig 1

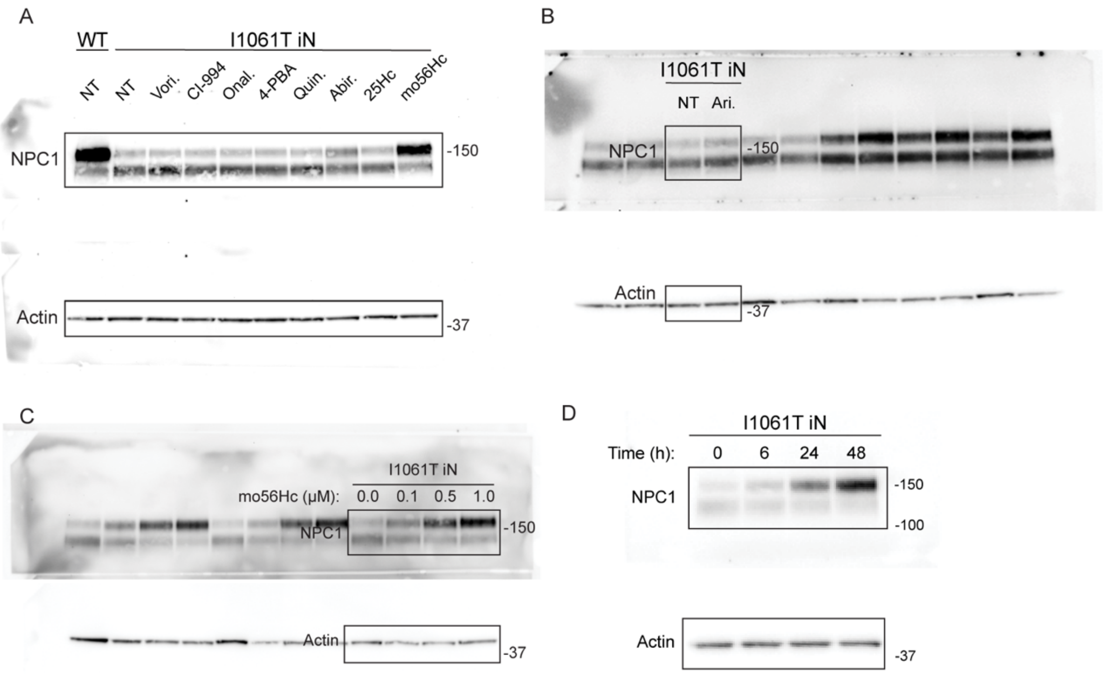
A
WT
I1061T iN
NT
NT
Vori.
CI-994
Onal.
4-PBA
Quin.
Abir.
25Hc
mo56Hc
NPC1
-150
Actin
-37
B
I1061T iN
NT
Ari.
NPC1
-150
Actin
-37
C
I1061T iN
mo56Hc (µM):
0.0
0.1
0.5
1.0
NPC1
-150
Actin
-37
D
I1061T iN
Time (h):
0
6
24
48
NPC1
-150
-100
Actin
-37

Fig 2

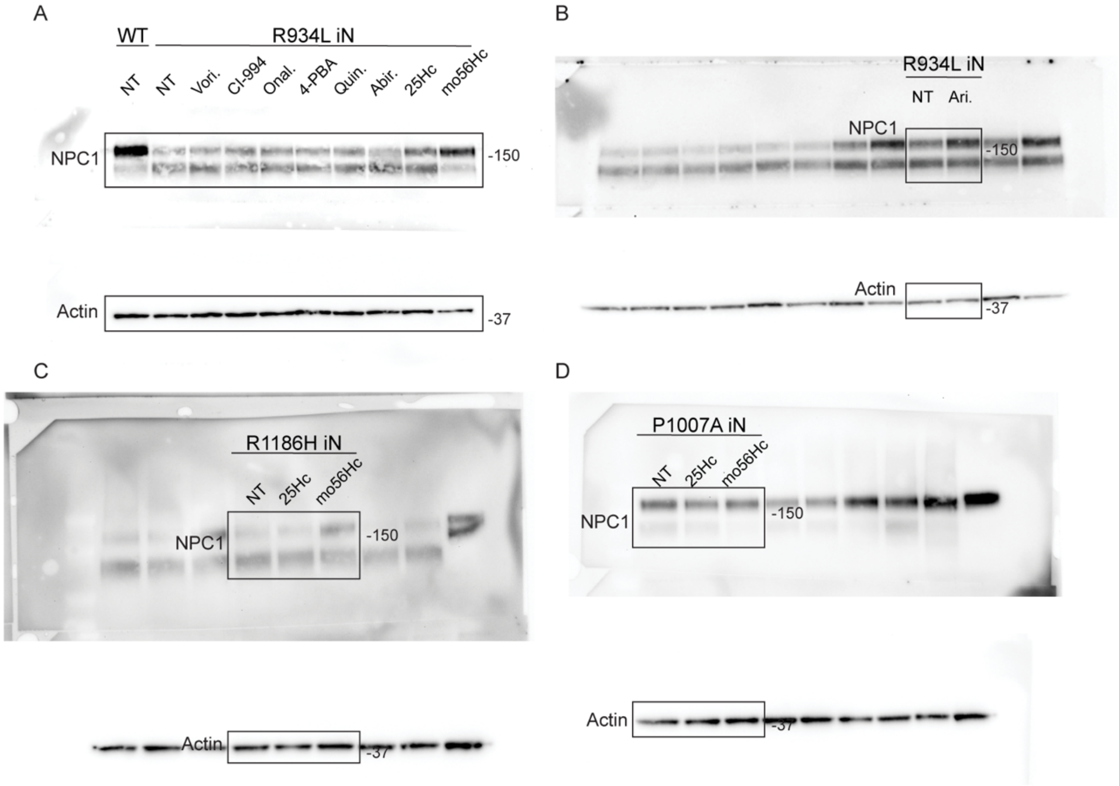
A
WT
R934L iN
NT
NT
Vori.
CI-994
Onal.
4-PBA
Quin.
Abir.
25Hc
mo56Hc
NPC1
-150
Actin
-37
B
R934L iN
NT
Ari.
NPC1
-150
Actin
-37
C
R1186H iN
NT
25Hc
mo56Hc
NPC1
-150
Actin
-37
D
P1007A iN
NT
25Hc
mo56Hc
NPC1
-150
Actin
-37

Fig 4

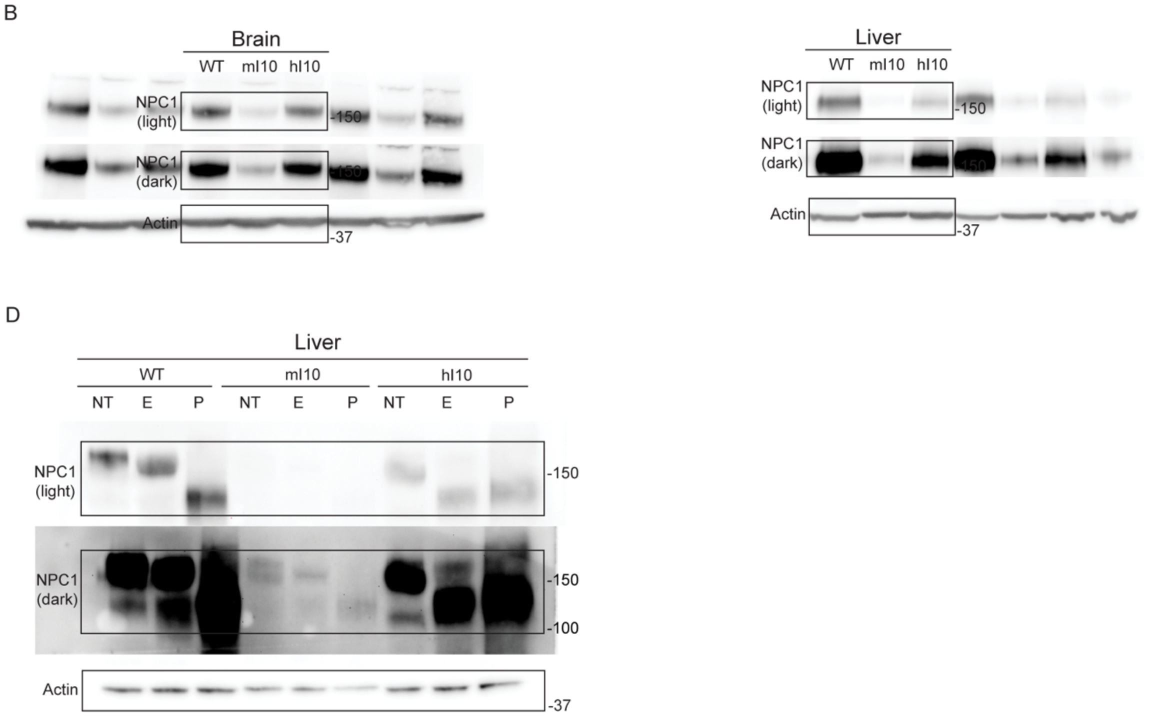
B
Brain
WT
mI10
hI10
NPC1 (light)
-150
NPC1 (dark)
-150
Actin
-37
Liver
WT
mI10
hI10
NPC1 (light)
-150
NPC1 (dark)
-150
Actin
-37
D
Liver
WT
mI10
hI10
NT
E
P
NT
E
P
NT
E
P
NPC1 (light)
-150
NPC1 (dark)
-150
-100
Actin
-37

Fig 6

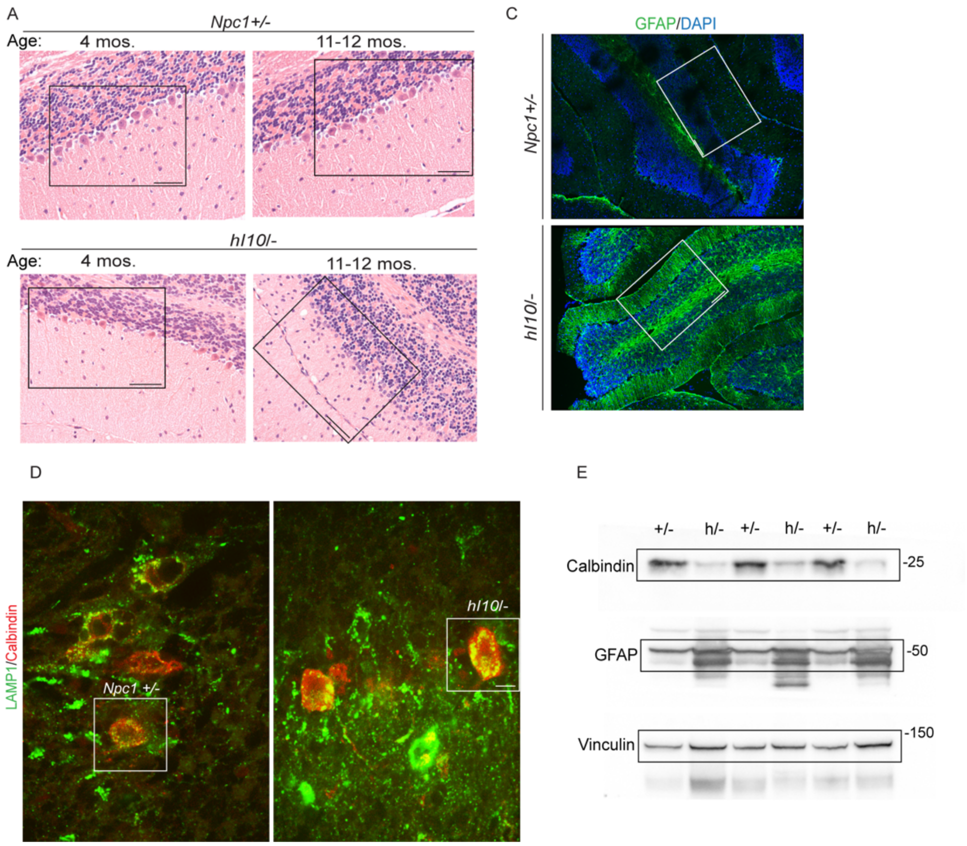
A
Npc1+/-
Age:
4 mos.
11-12 mos.
hl10/-
Age:
4 mos.
11-12 mos.
C
GFAP/DAPI
Npc1+/-
hl10/-
D
LAMP1/Calbindin
Npc1 +/-
hl10/-
E
+/-
h/-
+/-
h/-
+/-
h/-
Calbindin
-25
GFAP
-50
Vinculin
-150

Fig 8

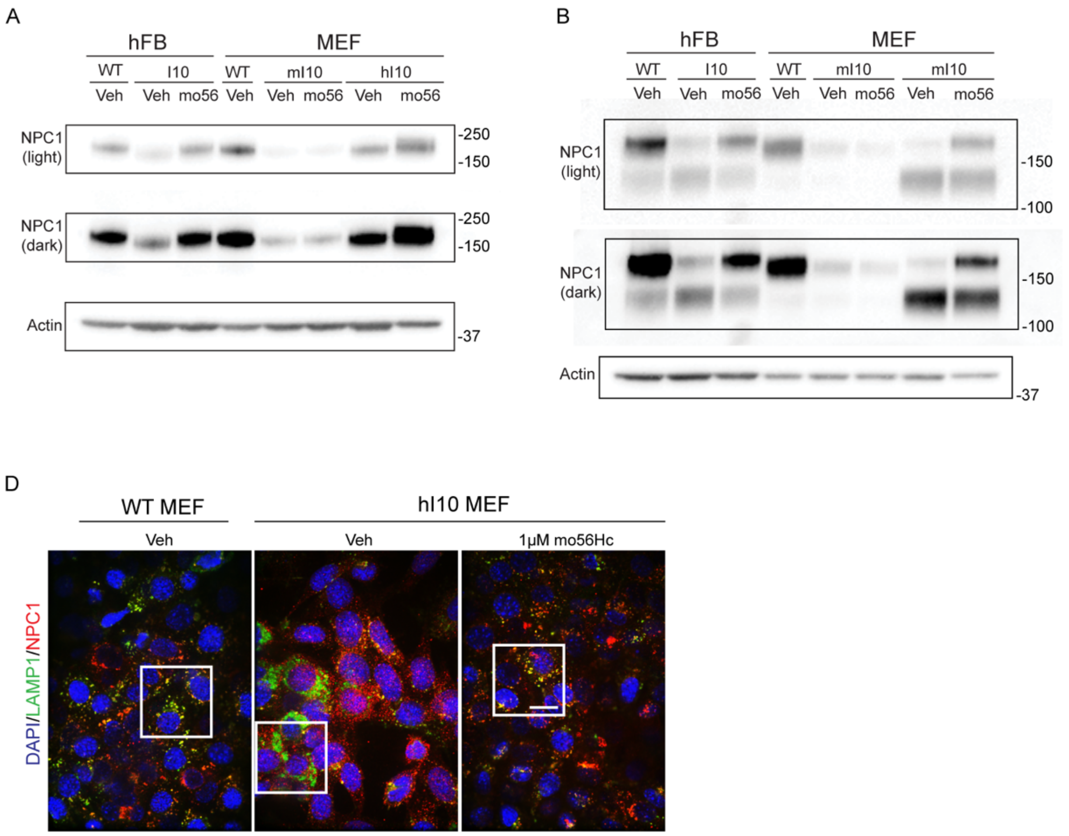
A
hFB
MEF
WT
I10
WT
mI10
hI10
Veh
Veh
mo56
Veh
Veh
mo56
Veh
mo56
NPC1 (light)
NPC1 (dark)
Actin
-250
-150
-37
B
hFB
MEF
WT
I10
WT
mI10
mI10
Veh
Veh
mo56
Veh
Veh
mo56
Veh
mo56
NPC1 (light)
NPC1 (dark)
Actin
-150
-100
-37
D
WT MEF
hI10 MEF
Veh
Veh
1μM mo56Hc
DAPI/LAMP1/NPC1

Fig S3

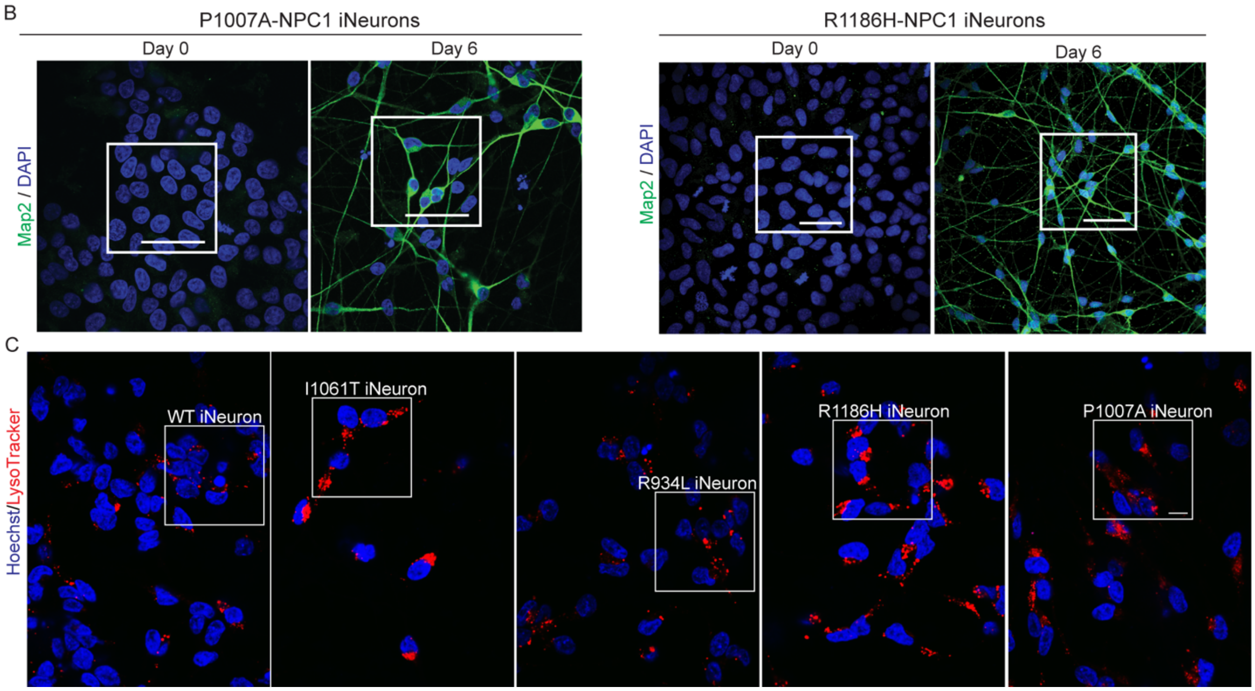
B
P1007A-NPC1 iNeurons
Day 0
Day 6
Map2 / DAPI
R1186H-NPC1 iNeurons
Day 0
Day 6
Map2 / DAPI
C
Hoechst/LysoTracker
WT iNeuron
I1061T iNeuron
R934L iNeuron
R1186H iNeuron
P1007A iNeuron

Fig S5

A

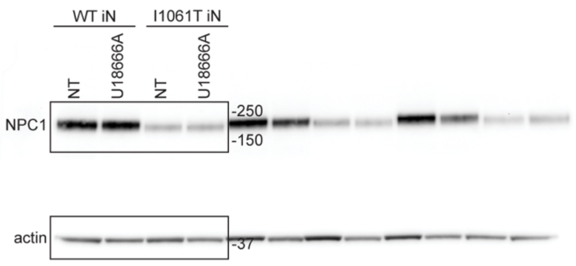
WT iN
I1061T iN
NT
U18666A
NT
U18666A
NPC1
-250
-150
actin
-37

B

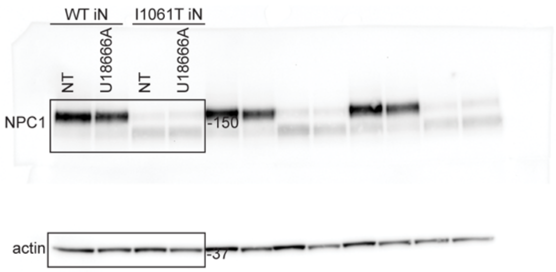
WT iN
I1061T iN
NT
U18666A
NT
U18666A
NPC1
-150
actin
-37

C

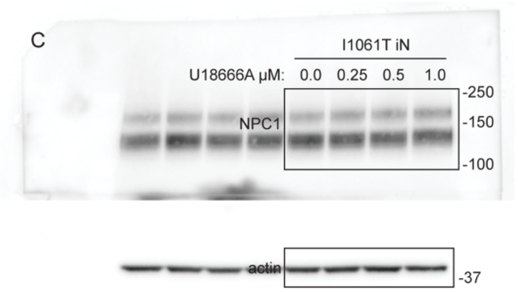
I1061T iN
U18666A μM: 0.0 0.25 0.5 1.0
-250
-150
NPC1
-100
actin
-37

Fig S6

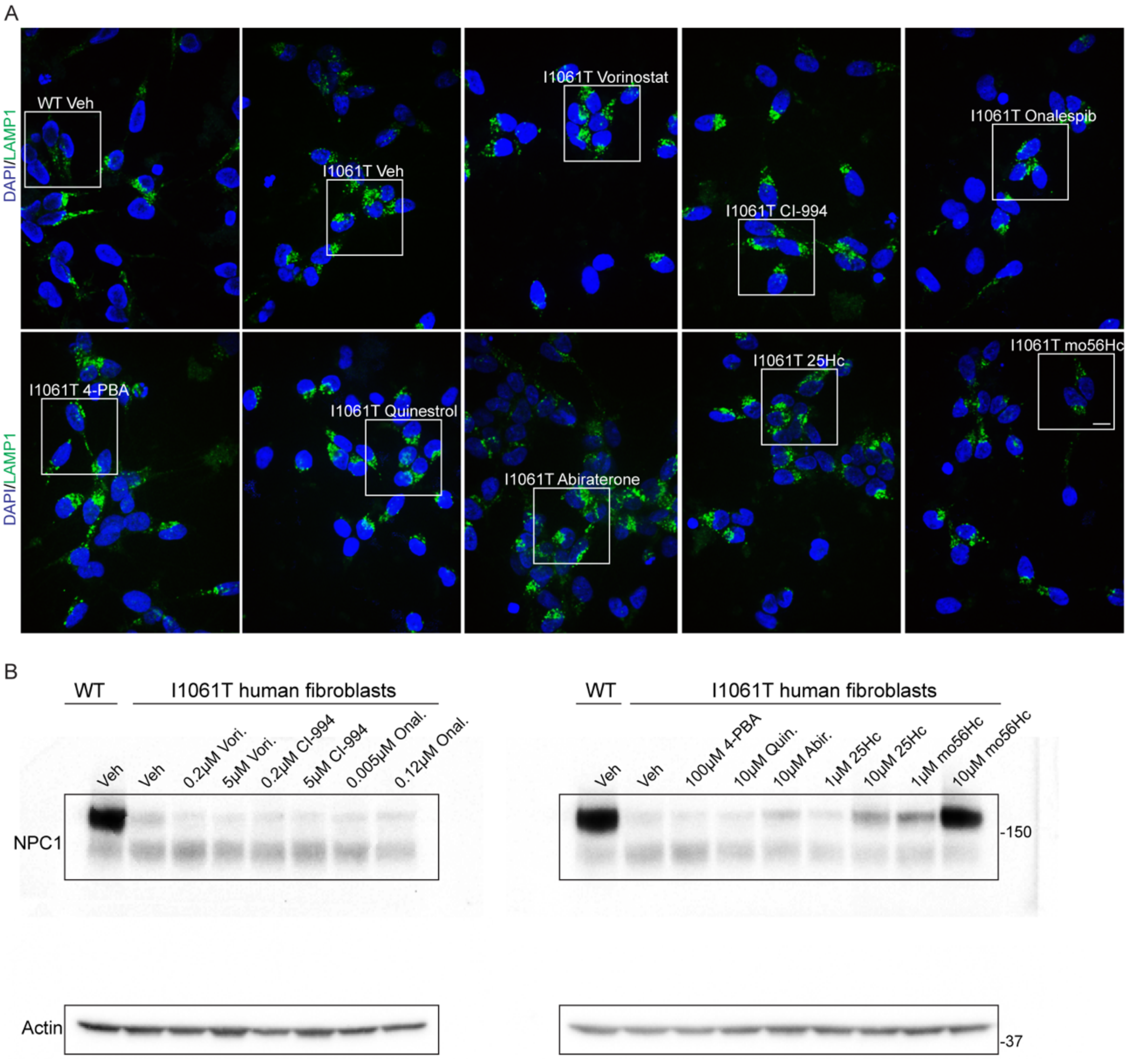
A
DAPI/LAMP1
WT Veh
I1061T Veh
I1061T Vorinostat
I1061T CI-994
I1061T Onalespib
I1061T 4-PBA
I1061T Quinestrol
I1061T Abiraterone
I1061T 25Hc
I1061T mo56Hc
B
WT
I1061T human fibroblasts
Veh
Veh
0.2μM Vori.
5μM Vori.
0.2μM CI-994
5μM CI-994
0.005μM Onal.
0.12μM Onal.
100μM 4-PBA
10μM Quin.
10μM Abir.
1μM 25Hc
10μM 25Hc
1μM mo56Hc
10μM mo56Hc
NPC1
Actin
-150
-37

Fig S9

B

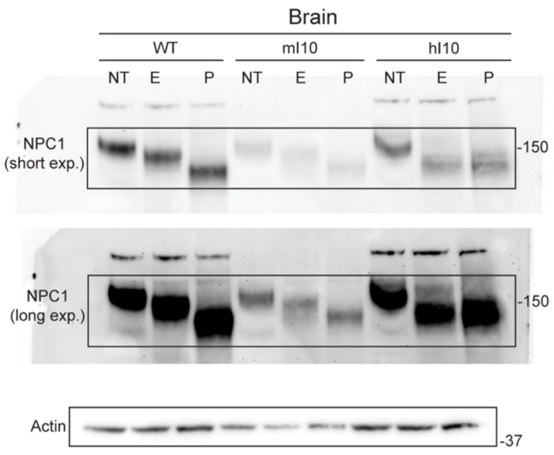

Brain
WT
mI10
hI10
NT
E
P
NT
E
P
NT
E
P
NPC1 (short exp.)
-150
NPC1 (long exp.)
-150
Actin
-37

Fig S10

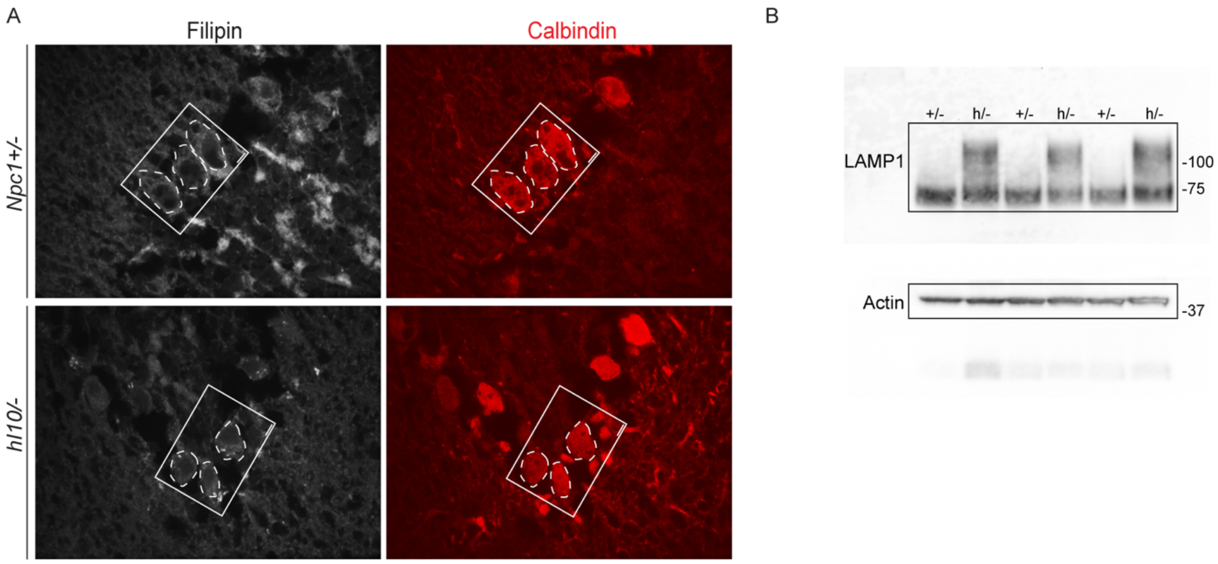

Fig S11

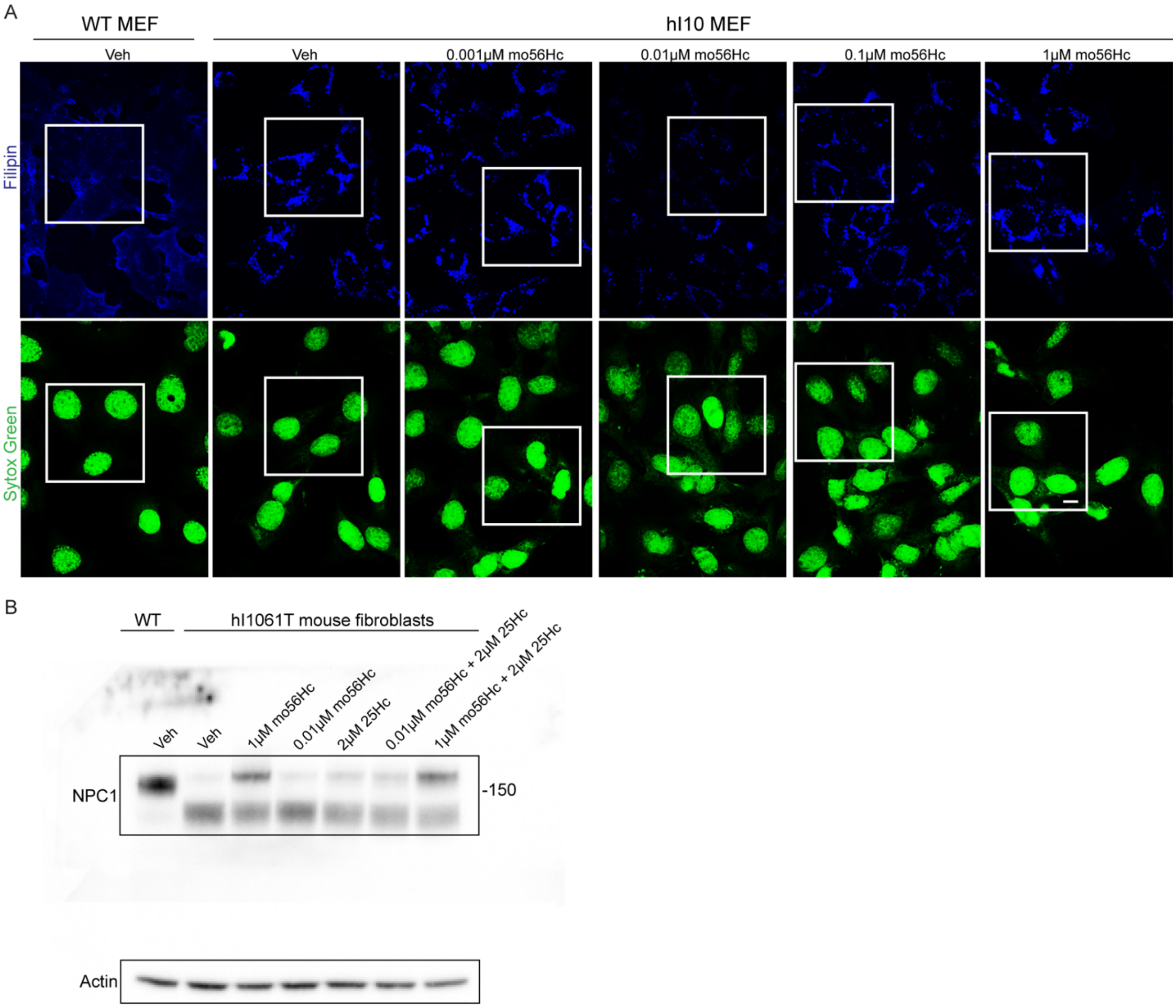
A
WT MEF
hI10 MEF
Veh
Veh
0.001μM mo56Hc
0.01μM mo56Hc
0.1μM mo56Hc
1μM mo56Hc
Filipin
Sytox Green
B
WT
hI1061T mouse fibroblasts
Veh
Veh
1μM mo56Hc
0.01μM mo56Hc
2μM 25Hc
0.01μM mo56Hc + 2μM 25Hc
1μM mo56Hc + 2μM 25Hc
NPC1
-150
Actin
